# Serum phosphorylated tau protein 181 and neurofilament light chain in cognitively impaired heart failure patients

**DOI:** 10.1186/s13195-022-01087-4

**Published:** 2022-10-10

**Authors:** Jan Traub, Markus Otto, Roxane Sell, Dennis Göpfert, György Homola, Petra Steinacker, Patrick Oeckl, Caroline Morbach, Stefan Frantz, Mirko Pham, Stefan Störk, Guido Stoll, Anna Frey

**Affiliations:** 1 Department of Internal Medicine I, University Hospital Würzburg, Oberdürrbacherstraße 6, 97080 Würzburg, Germany; 2 Comprehensive Heart Failure Center, University and University Hospital Würzburg, Am Schwarzenberg 15, Würzburg, 97078 Germany; 3 Department of Neurology, University Hospital Ulm, Ulm, Germany; 4 Department of Neurology, University Hospital Halle-Wittenberg, Halle, Germany; 5 Department of Psychiatry, Psychosomatics and Psychotherapy, University Hospital Würzburg, Würzburg, Germany; 6 Department of Neuroradiology, University Hospital Würzburg, Würzburg, Germany; 7 German Center for Neurodegenerative Diseases (DZNE e.V.), Ulm, Germany; 8 Department of Neurology, University Hospital Würzburg, Würzburg, Germany

**Keywords:** Alzheimer’s dementia, Heart failure, Cognitive impairment, Neurofilament light chain, Phosphorylated tau protein, Renal function, Age

## Abstract

**Background:**

Chronic heart failure (HF) is known to increase the risk of developing Alzheimer’s dementia significantly. Thus, detecting and preventing mild cognitive impairment, which is common in patients with HF, is of great importance. Serum biomarkers are increasingly used in neurological disorders for diagnostics, monitoring, and prognostication of disease course. It remains unclear if neuronal biomarkers may help detect cognitive impairment in this high-risk population. Also, the influence of chronic HF and concomitant renal dysfunction on these biomarkers is not well understood.

**Methods:**

Within the monocentric Cognition.Matters-HF study, we quantified the serum levels of phosphorylated tau protein 181 (pTau) and neurofilament light chain (NfL) of 146 extensively phenotyped chronic heart failure patients (aged 32 to 85 years; 15.1% women) using ultrasensitive bead-based single-molecule immunoassays. The clinical work-up included advanced cognitive testing and cerebral magnetic resonance imaging (MRI).

**Results:**

Serum concentrations of NfL ranged from 5.4 to 215.0 pg/ml (median 26.4 pg/ml) and of pTau from 0.51 to 9.22 pg/ml (median 1.57 pg/ml). We detected mild cognitive impairment (i.e., *T*-score < 40 in at least one cognitive domain) in 60% of heart failure patients. pTau (*p* = 0.014), but not NfL, was elevated in this group. Both NfL (*ρ* = − 0.21; *p* = 0.013) and pTau (*ρ* = − 0.25; *p* = 0.002) related to the cognitive domain visual/verbal memory, as well as white matter hyperintensity volume and cerebral and hippocampal atrophy. In multivariable analysis, both biomarkers were independently influenced by age (*T* = 4.6 for pTau; *T* = 5.9 for NfL) and glomerular filtration rate (*T* = − 2.4 for pTau; *T* = − 3.4 for NfL). Markers of chronic heart failure, left atrial volume index (*T* = 4.6) and NT-proBNP (*T* = 2.8), were further cardiological determinants of pTau and NfL, respectively. In addition, pTau was also strongly affected by serum creatine kinase levels (*T* = 6.5) and ferritin (*T* = − 3.1).

**Conclusions:**

pTau and NfL serum levels are strongly influenced by age-dependent renal and cardiac dysfunction. These findings point towards the need for longitudinal examinations and consideration of frequent comorbidities when using neuronal serum biomarkers.

**Supplementary Information:**

The online version contains supplementary material available at 10.1186/s13195-022-01087-4.

## Background

Alzheimer’s dementia (AD) is a growing medical and health economic challenge with a rapidly increasing incidence due to aging and already affects approximately 50 million people worldwide [1]. Whereas a strict dividing line was once conceptually drawn between AD and vascular dementia, it is now widely accepted that cardio- and cerebrovascular risk factors significantly increase the likelihood of developing AD [2, 3]. In addition to stroke, chronic heart failure (HF) in particular predisposes to the development of AD, as shown in population-based studies [4, 5]. HF is a common and serious consequence of many cardiac diseases and carries a grave prognosis [6]. More than 50% of chronic HF patients show mild cognitive impairment interfering with their daily activities and affecting their drug compliance [7].

The ability of highly sensitive immunoassays to detect ultra-low serum levels of neuronal biomarkers has made them even more useful for prognostic or diagnostic purposes in numerous neurodegenerative diseases and dementia [8]. In particular, serum neurofilament light chain (NfL), a structural neuronal intermediate filament, has evolved as a promising biomarker in pre-clinical and clinical dementia [9–11]. Serum NfL is closely associated with brain volume loss and cognitive deficits in general [12]. In contrast, tau protein, which is expressed abundantly in neurons and—at lower levels—in astrocytes and oligodendrocytes, promotes microtubule assembly and stability [13]. Abnormal hyperphosphorylation leads to increased levels of phosphorylated tau 181 (pTau) in cerebral spinal fluid and serum. pTau emerged as a plasma and serum biomarker of cerebral tau and amyloid pathologies, which are hallmarks of AD [14, 15].

At present, it is unclear, whether NfL or pTau serum levels indicate cognitive involvement in non-neurological diseases and if they are affected by internistic co-morbidity. In the present study of patients with chronic stable HF, 60% showed (mild) cognitive impairment, while the diagnosis of manifest dementia was an exclusion criterion at study entry [16, 17]. Thus, the Cognition.Matters-HF study offers the opportunity to test whether serum concentrations of both biomarkers may relate to subtle cognitive impairment and brain degeneration in this cohort, which is at high risk for developing manifest dementia.

## Methods

### Study design

We made use of the clinical data and biomaterials collected in the course of the prospective, monocentric Cognition.Matters-HF study. Its design and applied methodology have been reported in detail previously [16, 17]. The study recruited adult patients with chronic stable HF according to the then-current guidelines of the European Society of Cardiology [18]. Patients with apparent neurological or psychiatric disease, history of clinical stroke, or carotid artery stenosis over 50% were not eligible. The selection criteria are summarized in Supplemental Table S1. Cognition.Matters-HF was conducted in compliance with the Declaration of Helsinki and approved by the local ethics committee (#245/10) [17].

### Clinical evaluation

Physical examination, electrocardiography, echocardiography, and 6-min walk test were performed according to the standard operating procedures by the trained staff (for details, refer to Supplemental Methods) [16, 17]. The neurological evaluation included extensive clinical examination. Psychological testing was performed between 9 a.m. and 11 a.m. using a comprehensive test battery based on a taxonomy of attention dimensions (summarized in Supplemental Table S2). *T*-standardized output values accounting for the modifying effect of age, gender, and educational level were reported with a mean of 50 and a standard deviation of 10.

### Cerebral magnetic resonance imaging

Brain MRI was performed at a 3-T scanner (Siemens MAGNETOM Trio, Siemens Healthcare, Erlangen, Germany) as described previously [17]. Briefly, the applied MRI protocol enabled the estimation of global and regional measures of brain structure degeneration by visual rating (Supplemental Table S3). The visual rating of cerebral atrophy ranged on a scale from 1 to 8. For the assessment of medial temporal lobe atrophy, Scheltens score ranging from 0 (normal) to 4 (severe atrophy) was applied, and the mean scores of both sides (left and right) were reported.

### Laboratory analysis

Non-fasting venous blood samples were collected for routine clinical chemistry investigations at the certified facility of the University Hospital Würzburg. Participants were positioned seated for at least 5 min before puncture. Serum samples were processed immediately, i.e., remained at room temperature for 30 min and were centrifuged for 10 min at 2000 × *g*. The serum was aliquoted in dedicated fluid tissue tubes (Micronic, Lelystad, The Netherlands) and stored in the standardized interdisciplinary biomaterial bank at − 80 °C until analysis [19]. Serum NfL was measured using 5 (ProteinSimple™; San Jose, CA, USA) and pTau using the Simoa pTau-181 advantage kit (103377; Quanterix™, Billerica, MA, USA) on a Simoa HD-1 Analyzer instrument (Quanterix™, Billerica, MA, USA) in accordance with the manufacturer’s instructions. These measurements were performed blinded to the patients’ other results at the Department for Neurology of the University Hospital Ulm [9]. The inter-assay coefficient of variability for the human NF-L Ella instrument kit was 9.6% for high and 21.6% for low serum quality control, with a lower limit of quantification of 5.4 pg/ml. The intra-assay coefficient of variability of this assay has been validated elsewhere [20]. For the Simoa pTau-181 advantage kit, the inter-assay coefficient of variability was 7.0% for high and 11.2% for low-quality control. The intra-assay coefficient of variability was 1.4–9.3% for high- and 9.5–13.8% for low serum quality control. The lower limit of quantification for pTau was 0.222 pg/ml. Samples were measured in single determination and not grouped by cognitive parameters. For one sample, the NfL value was below the lower limit of quantification. Here, for the statistical calculations, the value of 5.4 pg/ml was used.

### Data analysis

Statistical analysis was performed using the statistical software SPSS (version 26). To test for normal distribution, we used the Shapiro-Wilk test. Variables were natural log normalized if required. The few missing values (less than 1% missing) were imputed by the mean value of the respective variable. For nominal and ordinal data, the chi-squared test or Fisher’s exact test was used, according to the nature of the data. For correlation analysis, the Spearman rho coefficient was computed. To identify the metric correlates of NfL and pTau levels, univariable linear regression analysis was used and a trend test across quartiles was reported. All tests were performed 2-sided.

When identifying determinants of NfL and pTau, to reduce the overoptimism introduced by multiple testing for the 79 clinical parameters that were used for analysis, a false discovery rate approach using the Benjamini–Hochberg procedure was applied. Thus, identified correlates were then included in univariable regression models. Only significant parameters were included in a multivariable model. Here, we analyzed the correlates using an “enter” approach. Second, we fed the remaining correlates into another regression model, which then was reduced to the final model through backward elimination and reproduced by forward entry. The explained variance of a model was indicated by the coefficients of determination (*R*^2^). To address collinearity, we calculated the variance inflation factors for all parameters of the multivariable models [21]. It represents the ratio of the variance of a model’s regression coefficients divided by the variance of a single coefficient. As all inflation factors were < 5, multi-collinearity was considered unlikely.

Using the SPSS PROCESS macro, a simple mediation was performed to analyze whether the total effect (*c*) of identified correlates was (partly) mediated by one another. This was considered the case, when the effect on the mediator (*a*) and the effect of the mediator (*b*) were significant. Partial mediation was claimed, when both direct (*c*′) and indirect (*ab*) effects were significant.

## Results

### Patient characteristics

Serum samples from 146 HF patients exhibiting no focal neurological deficits were available for analysis. As described in Table 1 and reported earlier [17], their age ranged from 32 to 85 years with a mean of 63.8 ± 10.8 years and 15.1% of the participants were women. The mean left ventricular ejection fraction was 42.5 ± 8.2% (16.4% had a preserved ejection fraction defined as ≥ 50%), and 72% of the patients were in New York Heart Association (NYHA) functional class II or III. Ischemia was the predominant underlying cause of HF (65%), and 84% of all patients received optimal HF therapy according to then-current guidelines at the study start [18]. The mean estimated glomerular filtration rate (eGFR) was 66.5 ± 19.4 ml/min/1.73 m^2^, and 35.6% had a chronic kidney disease of at least grade 3 (defined as eGFR < 60 ml/min/1.73 m^2^).

**Table 1:** Patient characteristics according to cognitive impairment (*T*-score < 40 in at least one domain)

	All patients, ***n*** = 146	No impairment, ***n*** = 58 (39.7%)	Cognitive impairment, ***n*** = 88 (60.3%)	***p***-value
**Clinical parameters**				
Age (years)	63.8 (10.8)	62.1 (11.4)	64.9 (10.3)	0.129
Female sex	22 (15.1%)	6 (10.3%)	16 (18.2%)	0.195
Body mass index (kg/m^2^)	29.1 (5.2)	29.5 (6.4)	28.8 (4.3)	0.451
Systolic blood pressure (mmHg)	138.1 (19.9)	134.7 (19.3)	140.4 (20.1)	0.090
Diastolic blood pressure (mmHg)	81.0 (11.0)	80.0 (11.4)	81.8 (10.9)	0.354
Heart rate (beats per minute)	64.5 (10.4)	63.8 (10.8)	65.1 (10.3)	0.452
Diabetes mellitus type II^a^	42 (28.8%)	16 (27.6%)	26 (29.5%)	0.798
Arterial hypertension^b^	116 (79.5%)	44 (75.9%)	72 (81.8%)	0.383
Hyperlipidemia^c^	105 (71.9%)	45 (77.6%)	60 (68.2%)	0.216
(Former) smoking	88 (60.3%)	41 (70.7%)	47 (53.4%)	**0.037**
Apparent dementia	0 (0.0%)	–	–	–
Atrial fibrillation/atrial flutter	33 (22.6%)	13 (22.4%)	20 (22.7%)	0.965
Coronary artery disease	100 (68.5%)	38 (65.5%)	62 (70.5%)	0.530
History of myocardial infarction	80 (54.8%)	32 (55.2%)	48 (54.5%)	0.941
History of coronary artery bypass grafting	36 (24.7%)	13 (22.4%)	23 (26.1%)	0.610
Peripheral artery disease	14 (9.6%)	6 (10.3%)	8 (9.1%)	0.801
Angiotensin-converting enzyme inhibitor	86 (58.9%)	35 (60.3%)	51 (58.0%)	0.774
Beta-blocker	131 (89.7%)	51 (87.9%)	80 (90.9%)	0.562
Aldosteron antagonist	54 (37.0%)	20 (34.5%)	34 (38.6%)	0.611
Diuretics	80 (54.8%)	29 (50.0%)	51 (58.0%)	0.345
Acetylsalicylic acid	80 (54.8%)	28 (48.3%)	52 (59.1%)	0.199
Coumadin or novel oral anticoagulant	45 (30.8%)	16 (27.6%)	28 (31.8%)	0.586
**Parameters of cardiac and renal dysfunction**				
Left ventricular ejection fraction (%)	42.5 (8.2)	41.7 (7.8)	43.0 (8.4)	0.337
Left atrial volume index (ml/min)	42.0 (17.5)	40.6 (14.1)	42.6 (18.9)	0.481
6-min walk test (m)	391.5 (99.3)	417.2 (97.6)	375.0 (97.5)	**0.014**
NT-proBNP (pg/ml)	1330 (2041)	1192 (2409)	1422 (1764)	0.515
eGFR (ml/min)	66.5 (19.4)	69.4 (19.5)	64.6 (19.2)	0.149
**Cognitive test battery**				
Intensity of attention (*T*-score)	41.8 (7.5)	47.2 (5.6)	38.3 (6.5)	**< 0.001**
Visual/verbal memory (*T*-score)	45.3 (6.2)	50.3 (6.7)	42.0 (6.9)	**< 0.001**
Executive functions (*T*-score)	45.4 (5.3)	47.8 (4.6)	43.9 (5.2)	**< 0.001**
Selectivity of attention (*T*-score)	45.3 (7.9)	46.5 (5.7)	44.4 (6.4)	0.052
Working memory (*T*-score)	46.2 (8.5)	49.3 (8.0)	44.2 (8.3)	**< 0.001**
Visual/verbal fluency (*T*-score)	44.8 (7.1)	47.6 (5.6)	43.0 (7.6)	**< 0.001**
**Magnetic resonance imaging**				
WMH volume (mm^3^)	4.36 (6.20)	3.88 (4.58)	4.67 (7.07)	0.452
Cerebral atrophy score (1–8)	3.25 (1.26)	3.15 (1.38)	3.31 (1.19)	0.447
Hippocampal atrophy score (0–4)	2.04 (0.90)	1.88 (0.86)	2.14 (0.91)	0.085
**Serum biomarkers**				
Neurofilament light chain (pg/ml)	34.0 (27.8)	30.7 (23.7)	36.2 (30.1)	0.247
Phosphorylated tau protein (pg/ml)	1.69 (1.33)	1.66 (0.88)	2.16 (1.53)	**0.014**

*eGFR* estimated glomerular filtration rate, *WMH* white matter hyperintensity

^a^History of diabetes mellitus type II or HbA1c > 6.5%

^b^Sitting blood pressure > 140/90 mmHg or history of hypertension before the onset of heart failure

^c^Hyperlipidemia or statin treatment

### Range of neuronal biomarkers

Serum concentrations of NfL ranged from 5.4 to 215.0 pg/ml with a median of 26.4 pg/ml (quartiles 16.7, 42.0). For pTau, the range was 0.51 to 9.22 pg/ml with a median of 1.57 (1.09, 2.41) pg/ml. The levels of both biomarkers did not differ between the sexes. The distribution of biomarkers is detailed in histograms in Fig. S1. Of note, NfL and pTau correlated to one another significantly (*ρ* = 0.57; *p* < 0.001).

### NfL and pTau are related to memory function and brain atrophy

**Fig. 1:**
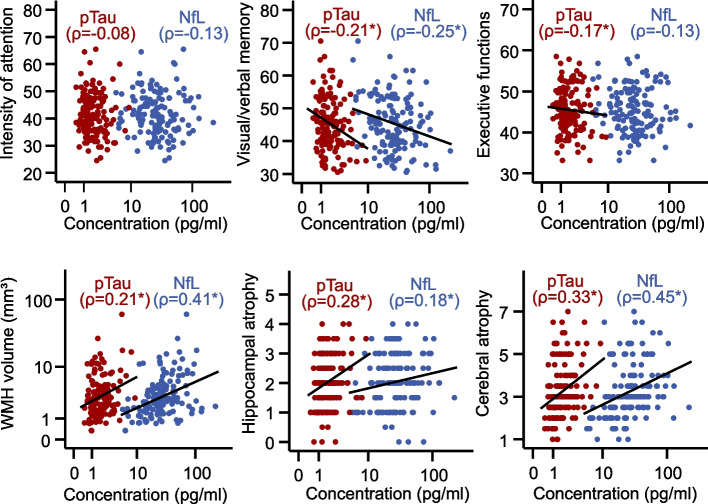
Scatter plot of NfL and pTau with cognitive domains and MRI parameters. Age-adjusted *T*-scores of cognitive domains are shown. Spearman correlation coefficient (*ρ*) is shown. WMH, white matter hyperintensity (*n* = 146; *p < 0.05)

As depicted in Table 1, patients with cognitive impairment (defined as age-adjusted *T*-score < 40 in at least one domain) had higher levels of pTau, which was not the case for NfL. Also, they had a lower 6-min walking test distance as a measure of chronic HF, while age did not differ between the groups. When examining the distinct cognitive domains in more detail (Fig. 1; Supplemental Table S4), both NfL and pTau significantly correlated to the cognitive domain visual/verbal memory while only pTau related to the selectivity of attention. Furthermore, both biomarkers positively correlated with volume of WMH and cerebral and hippocampal atrophy (quantified by Scheltens score).

### Age, renal function, and severity of heart failure predict NfL and pTau serum levels

Next, to identify if clinical parameters affect the serum levels of NfL and pTau, trend tests across quartiles per biomarker were performed (Supplemental Tables S5 and S6). After adjustment for multiple testing and confirmation in univariate regression (Tables 2 and 3), the following interrelations emerged: Patients with higher NfL concentrations were older and had lower estimated glomerular filtration rate (eGFR), larger left atrial volume index (LAVI), lower levels of mean corpuscular hemoglobin concentration and thrombocytes, but higher levels of urea, NT-proBNP, hemoglobin A1c, and uric acid. Furthermore, they had higher systolic pressure, reduced 6-min walking distance, and lower levels of hemoglobin and alanine aminotransferase. High NfL levels were also associated with a longer duration of HF and a more frequent intake of diuretics. Likewise, patients with higher pTau concentrations were older and had lower eGFR, larger left atrial volume index (LAVI), and increased right ventricular diameter. Additionally, they exhibited higher levels of creatine kinase, uric acid, urea, and NT-proBNP, as well as lower levels of thrombocytes, ferritin, mean corpuscular hemoglobin concentration, and alanine aminotransferase.

**Table 2:** Uni- and multivariable correlates of Ln (neurofilament light chain)

	Number	Separate models, univariable (enter)			Separate models, multivariable (enter)			Separate models, multivariable (backward)			Combined model, multivariable (enter)		
		*T*	*p*-value	*R*^2^	VIF	*T*	*p*-value	VIF	*T*	*p*	VIF	*T*	*p*-value
**Clinical parameters**					*R*^2^ = 0.53			*R*^2^ = 0.47			*R*^2^ = 0.48		
Age (years)	146	8.19	< 0.001	0.32	1.86	3.73	< 0.001	1.22	5.86	< 0.001	1.83	4.57	< 0.001
Estimated GFR (ml/min/1.73 m^2^)	146	− 7.58	< 0.001	0.29	2.18	− 2.78	0.006	1.45	− 3.44	0.001	1.55	− 3.10	0.002
Urea (mg/dl)	146	6.09	< 0.001	0.20	2.09	0.80	0.425						
NT-proBNP (pg/ml)	140	5.09	< 0.001	0.16	1.62	2.20	0.030	1.20	2.75	0.007	1.35	2.58	0.011
Mean corpuscular hemoglobin concentration (g/dl)	146	− 4.34	< 0.001	0.12	2.02	− 0.58	0.560						
Left-atrial volume index (ml/m^2^)	143	3.62	< 0.001	0.09	1.52	0.94	0.348						
Thrombocytes (10^3^/μl)	145	− 3.02	0.003	0.06	1.39	− 1.41	0.160						
Systolic blood pressure (mmHg)	145	3.25	0.001	0.07	1.37	1.58	0.116						
Diuretics (n/y)	146	− 4.73	< 0.001	0.13	1.37	− 1.93	0.055	1.18	− 2.07	0.051	1.21	− 1.97	0.057
6-min walking test distance (m)	138	− 3.34	0.001	0.08	1.55	1.90	0.060						
Hemoglobin (g/dl)	146	− 3.67	< 0.001	0.09	1.94	0.07	0.948						
Duration of heart failure (years)	145	2.60	0.010	0.05	1.23	0.74	0.461						
Alanine aminotransferase (U/l)	145	− 3.35	0.001	0.07	1.32	− 0.90	0.368						
Hemoglobin A1c (%)	145	2.11	0.037	0.03	1.22	1.28	0.202						
Uric acid (mg/dl)	146	2.08	0.039	0.03	1.36	− 1.79	0.076						
**MRI parameters**					*R*^2^ = 0.41			*R*^2^ = 0.41					
White matter hyperintensity volume (mm^3^)	146	3.70	< 0.001	0.30	1.06	2.84	0.005	1.06	2.90	0.004	1.15	1.11	0.267
Cerebral atrophy score (1–8)	146	4.37	< 0.001	0.34	1.17	3.33	0.001	1.06	3.69	< 0.001	1.63	0.23	0.816
Hippocampal atrophy score (0–4)	146	2.08	0.039	0.17	1.13	0.57	0.573						
**Cognitive domains**					*R*^2^ = 0.06			*R*^2^ = 0.06					
Intensity of attention (*T*-score)	146	− 0.83	0.407	0.01									
Visual/verbal memory (*T*-score)	146	− 3.12	0.002	0.06	1.00	− 3.12	0.002	1.00	− 3.12	0.002	1.13	− 0.63	0.529
Executive functions (*T*-score)	146	− 1.11	0.267	0.01									
Selectivity of attention (*T*-score)	146	− 1.34	0.181	0.01									
Working memory (*T*-score)	146	− 0.89	0.374	0.01									
Visual/verbal fluency (*T*-score)	146	− 0.25	0.805	0.00									

*GFR* glomerular filtration rate according to the MDRD formula, *VIF* variance inflation factor, *T T*-value here indicates the direction of the association and the relative weight of a variable in a model, *R*^*2*^ explained variance (by variable or model, respectively)

**Table 3:** Uni- and multivariable clinical correlates of Ln (phosphorylated tau protein)

	Number	Separate models, univariable (enter)			Separate models, multivariable (enter)			Separate models, multivariable (backward)			Combined model, multivariable (enter)		
		*T*	*p*-value	*R*^2^	VIF	*T*	*p*-value	VIF	*T*	*p*	VIF	*T*	*p*-value
**Clinical parameters**					*R*^2^ = 0.63			*R*^2^ = 0.58			*R*^2^ = 0.57		
Estimated GFR (ml/min/1.73 m^2^)	146	− 6.46	< 0.001	0.23	2.28	− 0.61	0.538	1.34	− 2.37	0.020	1.35	− 3.00	0.003
Age (years)	146	6.48	< 0.001	0.23	1.84	2.86	0.005	1.25	4.60	< 0.001	1.82	3.01	0.003
Urea (mg/dl)	146	6.11	< 0.001	0.21	2.24	0.61	0.538						
Left-atrial volume index (ml/m^2^)	143	5.31	< 0.001	0.17	1.93	1.99	0.049	1.09	4.57	< 0.001	1.10	4.60	< 0.001
Thrombocytes (10^3^/μl)	145	− 3.79	< 0.001	0.09	1.66	− 1.45	0.149						
Creatine kinase (U/l)	146	5.38	< 0.001	0.17	1.41	4.65	< 0.001	1.04	6.49	< 0.001	1.03	6.82	< 0.001
Duration of heart failure (years)	145	3.53	0.001	0.08	1.37	1.03	0.305						
Uric acid (mg/dl)	146	3.89	< 0.001	0.10	1.68	0.51	0.607						
NT-proBNP (pg/ml)	146	3.66	< 0.001	0.09	1.79	2.04	0.044						
Coumadin or novel oral anticoagulant (n/y)	146	− 4.06	< 0.001	0.10	1.74	0.45	0.653						
Creatine kinase, muscle-brain (U/l)	146	4.63	< 0.001	0.13	1.57	1.66	0.100						
Angiotensin-converting enzyme inhibitor (n/y)	146	0.25	0.805	0.00									
Mean corp. hemoglobin concentr. (g/dl)	146	− 2.29	0.024	0.04	1.96	0.51	0.605						
Ferritin (μg/l)	146	− 2.65	0.009	0.05	1.36	− 2.64	0.010	1.11	− 3.11	0.002	1.08	− 2.28	0.024
Diuretics (n/y)	146	− 3.40	0.001	0.07	1.41	− 0.13	0.894						
Basal right ventricular diameter (mm)	146	3.58	< 0.001	0.08	1.31	1.48	0.142						
Alanine aminotransferase (U/l)	146	− 2.28	0.024	0.04	1.41	0.06	0.952						
**MRI parameters**					*R*^2^ = 0.39			*R*^2^ = 0.39					
White matter hyperintensity volume (mm^3^)	146	2.96	0.004	0.24	1.06	2.05	0.043	1.06	2.05	0.043	1.15	0.12	0.905
Cerebral atrophy score (1–8)	146	3.91	<0.001	0.31	1.17	2.56	0.012	1.17	2.56	0.012	1.53	1.16	0.247
Hippocampal atrophy score (0–4)	146	3.41	0.001	0.27	1.13	2.17	0.032	1.13	2.17	0.032	1.21	− 1.09	0.278
**Cognitive domains**					*R*^2^ = 0.07			*R*^2^ = 0.06					
Intensity of attention (*T*-score)	146	− 0.86	0.389	0.01									
Visual/verbal memory (*T*-score)	146	− 3.13	0.002	0.06	1.18	− 2.48	0.014	1.00	− 3.13	0.002	1.13	− 1.09	0.883
Executive functions (*T*-score)	146	− 0.72	0.474	0.00									
Selectivity of attention (*T*-score)	146	− 2.13	0.035	0.03	1.18	− 1.04	0.302						
Working memory (*T*-score)	146	0.33	0.739	0.00									
Visual/verbal fluency (*T*-score)	146	0.17	0.863	0.00									

*GFR* glomerular filtration rate according to the MDRD formula, *VIF* variance inflation factor, *T T*-value here indicates the direction of the association and the relative weight of a variable in a model, *R*^*2*^ explained variance (by variable or model, respectively)

**Fig. 2:**
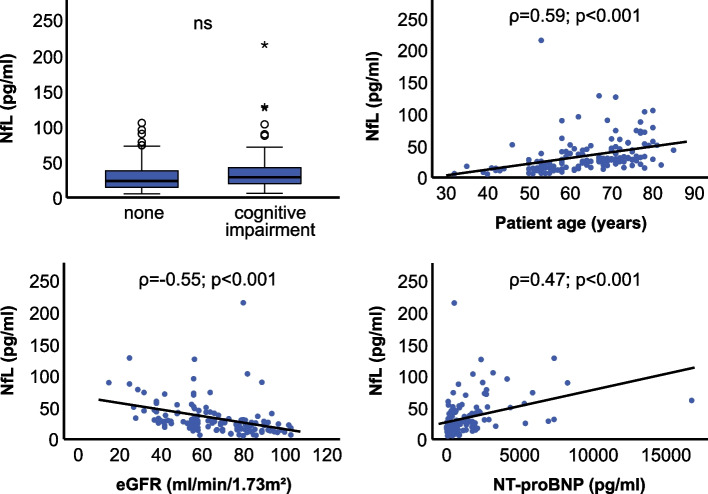
Boxplot and scatter plots of serum NfL. Spearman correlation coefficient (*ρ*) is shown. eGFR, estimated glomerular filtration rate (*n* = 146)

**Fig. 3:**
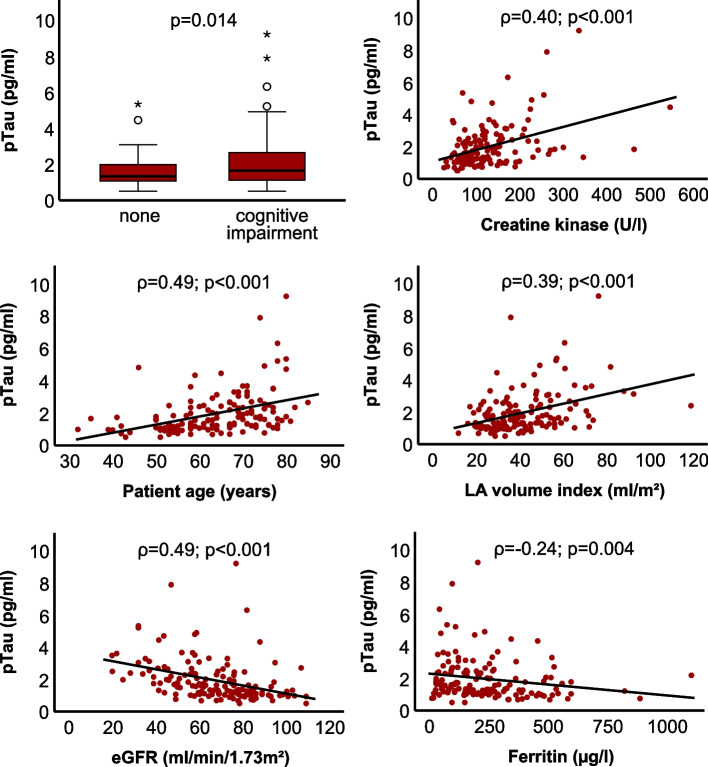
Boxplot and scatter plots of serum pTau. Spearman correlation coefficient (*ρ*) is shown. eGFR, estimated glomerular filtration rate; LA, left atrium (*n* = 146)

In a multivariable setting, which included all significant clinical correlates, age and eGFR emerged as strong independent clinical predictors of both biomarkers (Tables 2 and 3). This was also the case for the cardiac parameters NT-proBNP and LAVI, respectively. Furthermore, creatine kinase (CK) and ferritin levels remained strongly related to serum pTau in the final clinical model. Correlation plots of significant predictors are displayed in Figs. 2 and 3. Final clinical models of both NfL and pTau explained variance well, as indicated by *R*^2^ of 0.47 and 0.58, respectively.

### Cardiac and renal dysfunction partly mediate the effect of age on serum biomarkers

**Fig. 4:**
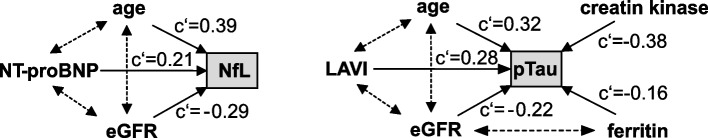
Visualization of direct and indirect effects on serum biomarkers. *c*′, direct effect in mediation analysis; eGFR, estimated glomerular filtration rate; LAVI, left atrial volume index

To discriminate if the identified predictors in the final, combined models have a direct or indirect (mediated) effect on the examined serum biomarkers, we performed a mediation analysis (Supplemental Tables S7 and S8). The effect of age on Ln(NfL) was partly mediated by eGFR and NT-proBNP. In line, eGFR and LAVI mediated the effect of age on Ln(pTau). Interestingly, the effect of CK on Ln(pTau) was not mediated by any of the mentioned parameters. These interrelations were finally visualized, as depicted in Fig. 4.

### Independent association between biomarkers and cognitive domains

Next, we analyzed if the above-identified clinical correlates of both biomarkers also related to age-adjusted memory *T*-score (Supplemental Tables S9 and S10), which was the case for renal function in the clinical model. In a thus combined model, NfL and pTau remained independent correlates of visual/verbal memory *T*-score. pTau also related independently to the selectivity of attention, even when clinical correlates of pTau were considered (Supplemental Table S11).

Finally, we tested if correlates of NfL and pTau relate to volume of WMH, and cerebral and hippocampal atrophy (Supplemental Tables S12, S13 and S14). Age and systolic blood pressure evolved as significant predictors of Ln(WMH volume) along with Ln(NfL) in a combined model. Age, eGFR, and NT-proBNP predicted global atrophy, while NT-proBNP and age were significant predictors of hippocampal atrophy.

## Discussion

This post hoc analysis in chronic stable HF patients of the well-phenotyped Cognition.Matters-HF study showed that mild cognitive impairment in this cohort is associated to subtle increases of pTau and NfL, especially regarding memory function. Serum levels of the neuronal biomarkers NfL and pTau were noticeably affected by numerous clinical routine parameters which are established indicators of comorbidity burden. Both NfL and pTau were strongly related to age and renal function and, in addition, were markedly affected by indicators of heart failure severity in the respective final model: NT-proBNP, an indicator of cardiac wall stress and congestion remained associated with NfL levels, and LAVI, an estimate of chronic elevation of cardiac filling pressures, as well as creatinine kinase, a marker of ongoing cardiac damage, remained associated with pTau. Of note, the absence of the association of left ventricular ejection fraction with both markers implies that our findings apply to the whole spectrum of HF, including reduced and preserved ejection fraction. These findings impact the interpretation of NfL and pTau in clinical research and care.

The current analysis was based on serum samples drawn at baseline from a thoroughly characterized cohort of chronic HF patients [17]. As our group has shown before, serum concentrations of NfL are comparable to measurements in plasma samples [9, 11]. For pTau, a very recent study similarly found comparable diagnostic performances and strong correlations between serum versus plasma [22]. A significant number of patients with HF in general [7] and within our cohort showed signs of cognitive impairment as 60% of patients exhibited deficits (*T*-score < 40) in at least one cognitive domain. We found independent associations of NfL and pTau to memory impairment in our cross-sectional analysis, even when clinical parameters were considered. This is in line with recently published relations of NfL and pTau to cognitive dysfunction in AD [23] and other neurological disorders [24].

There is strong epidemiological evidence that chronic HF predisposes to the development of AD [4, 5]. Accordingly, increased serum pTau in our HF cohort, which excluded patients with overt dementia at study entry, may indicate incipient AD development since serum/plasma pTau is considered a specific biomarker for AD [25]. This notion is based on observations showing that increased plasma pTau 181 correctly identified individuals who were amyloid β positive in positron emission tomography regardless of clinical diagnosis, and a positive association between higher levels of plasma pTau 181 levels and cortical tau protein deposition measured by F-18-Flortaucipir positron emission tomography [26]. Thus, serum/plasma pTau discriminated between AD and frontotemporal dementia. It will be interesting to sequentially measure serum pTau levels in HF patients to assess, whether these dynamics can predict clinical conversion from mild cognitive decline to overt AD.

With regard to morphologic brain changes, serum NfL and pTau correlated with WMH volume and hippocampal and cerebral atrophy in the univariable setting. In multivariable models including clinical parameters, only a slight association between Ln(NfL) and Ln(WMH volume) remained. The univariable associations of both biomarkers to visually rated global brain atrophy are in line with findings from patients with multiple sclerosis [27], frontotemporal dementia [9], AD [28], and also normal-aging individuals [12]. Furthermore, both biomarkers also positively correlated with the Scheltens score, which quantifies hippocampal atrophy, and has been related to the severity of cognitive symptoms in AD and Lewy body dementia [29, 30]. In patients with heart failure, hippocampal atrophy is a significant and independent predictor of memory impairment, executive dysfunction, and poor prognosis as reported earlier for the Cognition.Matters-HF study sample [17] and by others [31]. Finally, WMH are known to associate with diabetes, smoking, and hypertension and hence considered to be part of small vessel disease coinciding with demyelination, axonal loss, reduced glial density and atrophy [32], and accelerated cognitive decline [33]. Of note, our finding of an independent association of NfL with WMH lesion load in our cohort of HF patients devoid of focal neurological deficits confirms and substantiates an earlier study reporting such correlation in patients with AD [34].

We observed that clinical and laboratory factors profoundly influence NfL and pTau serum concentrations. Associations of NfL with aging are well known both in neurologically healthy subjects, whose brain atrophy during aging is thought to increase serum levels [12], as well as in neurological patients suffering from multiple sclerosis or stroke [35, 36]. While total tau protein in the serum increased with normal aging within a cognitively unaffected cohort [37], pTau values in the cerebrospinal fluid similarly showed a significant correlation with age [38]. Here, for the first time, we describe the association of serum pTau with increasing age and confirm age-dependent findings for NfL in a cohort of heart failure patients without neurological deficits. Interestingly, cardiac and renal dysfunction, but not MRI parameters partly mediated the effect of age on both biomarkers.

Previously, it has been reported that serum NfL levels are partially affected by renal function [36, 39], which was confirmed in our HF cohort, as eGFR negatively correlated with serum NfL. Moreover, we show that this also applies to serum pTau, which has not been reported so far, pointing towards an impaired clearance of these proteins.

Serum levels of creatine kinase evolved as another strong and independent predictor of pTau, but not NfL. This may be caused by the enzymatic activity of creatine kinase in the serum, leading to phosphorylation of tau protein. Furthermore, brain-derived BB isoforms of creatine kinase, diffusing into the serum upon neuronal damage, may increase total creatine kinase levels along with pTau levels. This novel finding can possibly be explained by the presence of tau protein in skeletal muscles [40].

The cross-sectional approach of the current study restricts the interpretation of NfL and pTau. However, the strength of this investigation lies in its extensive clinical work-up, including detailed cognitive testing that included five cognitive function domains instead of global tests. The high explained variance found for the overall models explaining serum NfL and pTau suggests that indeed relevant etiological factors were considered, thus stimulating further research in this direction.

## Conclusions

Overall, we found a slight elevation of serum pTau in chronic HF patients with mild cognitive impairment as a possible surrogate marker for impending development of AD. Furthermore, our results have implications for the clinical use of NfL and pTau serum levels as biomarkers for neurodegeneration and dementia such as AD, frontotemporal lobe degeneration, and amyotrophic lateral sclerosis. These conditions predominantly develop in the elderly, who are also frequently burdened by multiple comorbidities. While renal function and other laboratory parameters are regularly available through routine investigations, our study identifies chronic HF as a major confounder of serum NfL and/or pTau that has not been taken into consideration in the numerous previous reports using NfL and pTau as surrogate markers for neurodegeneration and/or dementia.

## Supplementary Information

**Additional file 1: Supplemental Methods. Table S1.** Inclusion and exclusion criteria. **Table S2.** Outcome of the cognitive test battery. **Table S3.** Imaging protocol and sequence parameters. **Table S4.** Correlation of biomarkers to cognitive domains and brain morphology. **Table S5.** Extended clinical characteristics of NfL quartiles. **Table S6.** Extended clinical characteristics of pTau quartiles. **Table S7.** Mediation analysis of parameters affecting serum levels of Ln(NfL). **Table S8.** Mediation analysis of parameters affecting serum levels of Ln(pTau). **Table S9.** Regression analysis of visual/verbal memory T-score with NfL. **Table S10.** Regression analysis of visual/verbal memory T-score with pTau. **Table S11.** Regression analysis of selectivity of attention T-score with pTau. **Table S12.** Regression analysis of cerebral atrophy. **Table S13.** Regression analysis of hippocampal atrophy. **Table S14.** Regression analysis of Ln (WMH volume). **Figure S1.** Histograms of serum NfL and pTau.

## Data Availability

The data that support the findings of this study are available from the corresponding author, upon reasonable request.

## Abbreviations

AD: Alzheimer’s dementia
eGFR: Estimated glomerular filtration rate
HF: Heart failure
LAVI: Left atrial volume index
NfL: Neurofilament light chain
pTau: Phosphorylated tau protein
WMH: White matter hyperintensities
