# Supplementary material for: Serum phosphorylated tau protein 181 and neurofilament light chain in cognitively impaired heart failure patients
Source: Alzheimers Res Ther. 2022 Oct 10;14:149. doi: 10.1186/s13195-022-01087-4 (PMC9549648; doi:10.1186/s13195-022-01087-4)
Supplement: Supplementary file 1 — Additional file 1: Supplemental Methods. Table S1. Inclusion and exclusion criteria. Table S2. Outcome of the cognitive test battery. Table S3. Imaging protocol and sequence parameters. Table S4. Correlation of biomarkers to cognitive domains and brain morphology. Table S5. Extended clinical characteristics of NfL quartiles. Table S6. Extended clinical characteristics of pTau quartiles. Table S7. Mediation analysis of parameters affecting serum levels of Ln(NfL). Table S8. Mediation analysis of parameters affecting serum levels of Ln(pTau). Table S9. Regression analysis of visual/verbal memory T-score with NfL. Table S10. Regression analysis of visual/verbal memory T-score with pTau. Table S11. Regression analysis of selectivity of attention T-score with pTau. Table S12. Regression analysis of cerebral atrophy. Table S13. Regression analysis of hippocampal atrophy. Table S14. Regression analysis of Ln (WMH volume). Figure S1. Histograms of serum NfL and pTau. [file 13195_2022_1087_MOESM1_ESM.docx]

**- SUPPLEMENTAL MATERIAL -**

**Serum phosphorylated tau 181 and neurofilament light chain in cognitively impaired heart failure patients**

**Supplemental Methods**

Cardiological evaluation page 2

Psychological Test Battery page 2

Neurological evaluation page 3

Ethical considerations page 3

**Supplemental Tables**

Table S1: Inclusion and exclusion criteria page 4

Table S2: Outcome of the cognitive test battery page 5

Table S3: Imaging protocol and sequence parameters page 6

Table S4: Correlation of biomarkers to cognitive domains and brain morphology page 7

Table S5: Extended clinical characteristics of NfL quartiles page 8

Table S6: Extended clinical characteristics of pTau quartiles page 12

Table S7: Mediation analysis of parameters affecting serum levels of Ln(NfL). page 16

Table S8: Mediation analysis of parameters affecting serum levels of Ln(pTau). page 17

Table S9: Regression analysis of visual/verbal memory T-score with NfL page 18 Table S10: Regression analysis of visual/verbal memory T-score with pTau page 19 Table S11: Regression analysis of selectivity of attention T-score with pTau page 20 Table S12**:** Regression analysis of cerebral atrophy. page 21 Table S13: Regression analysis of hippocampal atrophy page 22 Table S14: Regression analysis of Ln (WMH volume). page 23

**Supplemental Figures**

Figure S1: Histograms of serum NfL and pTau page 24

**Supplemental References** page 25

**Supplemental Methods**

**Cardiological evaluation**

A standard transthoracic echocardiographic examination was performed (GE, Vingmed, Horten, Norway) with the patient lying in a left-lateral position and recording a 3-channel electrocardiogram. Standard two-dimensional (2D) images and Doppler recordings were obtained according to current guidelines (1) and stored digitally. Care was taken to record three cardiac cycles, respectively. Offline measurements were performed using EchoPAC (version 112, GE, Horten, Norway). Left ventricular (LV) end-diastolic (LVEDD) and end-systolic (LVESD) and left atrial end-systolic dimensions (LAD) as well as end-diastolic wall thickness of the basal septum (IVSd) and the LV posterior wall (LVPWd) were measured in a parasternal LV long axis view. Left atrial end-systolic as well as LV end-diastolic (LVEDV) and end-systolic volumes (LVESV) were measured according to the Simpson´s biplane method in the apical 4- and 2-chamber views and LV ejection fraction (LVEF) was calculated: (LVEDV – LVESV) / LVEDV. Right ventricular basal dimension were obtained in the apical 4-chamber view. Peak velocity of early (E) and late (A) mitral valve inflow and deceleration time of E wave (DT) were measured using pulsed-wave (PW) Doppler with the acquisition window positioned at the mitral valve leaflet tips, and the E/A ratio was calculated. Early diastolic mitral annular relaxation velocity (e´) was obtained using PW tissue Doppler with the acquisition window positioned at the lateral annular site and E/e´ was calculated as non-invasive estimate of LV filling pressures. We screened for tricuspid valve regurgitation (TR) using color Doppler and in case of respective evidence, we traced TR velocity using continuous-wave Doppler and derived the maximal systolic trans-tricuspid pressure gradient. Diastolic function was graded according to the most current recommendations (2) integrating E, A, e´, left atrial volume indexed to body surface area, and the maximal systolic trans-tricuspid pressure gradient.

**Psychological Test Battery**

Intensity and selectivity of attention were tested by the Test Battery of Attentional Performance (TAP, Zimmermann & Fimm 2009, version 2.2) (3). To evaluate visual/verbal memory and working memory the Visual and Verbal Memory Test (2^nd^ edition, VVM2, Schellig & Schächtele 2009) (4), the Digit Span Forward and Block Tapping Span Forward tests of the Wechsler Memory Scale revised (WMS-R, Wechsler, 1987) (5) were applied. Visual/verbal fluency was analyzed using the Regensburger Word Fluency Test (RWT, Aschenbrenner, Tucher & Lange, 2000) (6) and the HAMASCH-5-Point-Test revised (H5PT-R, Haid, Martl & Schubert, 2004) (7). Premorbid intelligence was used as the control variable to exclude the influence of distinctive intelligence upon the results (8, 9). In addition, the HADS (Hospital Anxiety and Depression Scale) was applied to monitor depression (10, 11). Patient with overt depression were excluded and advised to consult a psychiatrist for further clinical examination. **Table S3** presents different subtests, their classification to the cognitive domains as well the chosen result parameters. Reliability of tests ranged between 0.60 and 0.99.

**Intensity of Attention**

To assess different attention processes the test battery of Attentional Performance (TAP, Zimmermann & Fimm 2009, version 2.2) was used. The *subtest Alertness* records reaction times with (*phasic alertness*) and without prepended acoustic signal (*tonic alertness*) and is thus been classified under the domain of intensity of attention.

**Selectivity of Attention**

The second parameter of attention – selectivity of attention – also was assessed by the test battery of Attentional Performance (TAP). Three different subtest were chosen: a subtest for response inhibition (*subtest “GoNoGo”*), in which patients have to show motor response in 1 of 2 conditions. A test with an acoustic and visual dual-task-condition (*subtest “Geteilte Aufmerksamkeit”*) records performance in divided attention. The third test assessing selectivity of attention as part of executive functions is a test to measure stimulus-response-interference effects (subtest *“Inkompatibilität”*) (3).

**Memory**

Visual and verbal short- and medium-term memory were assessed by the Visual and Verbal Memory Test 2nd edition (VVM2, Schellig & Schächtele 2009). The test is based on functions of the explicit memory. For visual memory a path in a map has to be learned, for verbal memory information has to be learned (names, numbers, etc.) from a continuous text (4). Learning content has to be reproduced immediately and after 30 minutes.

**Working Memory**

Digit span forward and block tapping span forward of the Wechsler Memory Scale revised (WMS-R, Wechsler, 1987) were used to assess visual and verbal working memory. For digit span the examiner reads a sequence of numbers with increasing lengths, which has to be repeated by the patient. For block tapping span the examiner taps a series of blocks and the patient is asked to repeat in the correct sequential order. Working memory is relevant for executive performance (5).

**Fluency**

As well verbal and visual fluency are relevant for executive performance. Verbal fluency and shifting was measured by the Regensburger Word Fluency Test (RWT, Aschenbrenner, Tucher & Lange, 2000). Within 2 minutes patients have to name as many german words as possible that begin with a specific letter (for example s) and in the following subtest with two alternate letters (for example g – r) (6). Visual fluency was quantified by the HAMASCH-5-Punkte-Test revised (H5PT-R, Haid, Martl & Schubert, 2004). On a sheet with identical rectangles with 5 points patients have to create within 3 minutes as many patterns as possible by drawing lines between at least 2 points (7).

**Neurological Examination**

Patients underwent a routine neurological examination to disclose focal neurological deficits due to previous stroke or other neurological comorbidities and standard extracranial and transcranial Doppler sonography to exclude middle or high-grade extracranial artery stenosis (12).

**Ethical considerations**

Cognition.Matters-HF is an observational monocentric study complying with GCP standards. Added patient risk is almost negligible and limited to complications of blood sampling and possible concerns of data privacy protection. The MRI examination has been immediately stopped in case of occurrence of claustrophobia. All parts of the study conform to the Declaration of Helsinki (World Medical Association. World Medical Association Declaration of Helsinki: ethical principles for medical research involving human subjects. Adopted by the 18th WMA General Assembly, Helsinki, Finland June 1964, last amended by the 59th WMA General Assembly, Seoul, October 2008 World Medical Association; 2008) and have been approved by the Ethics committee of the institution (study registration number 245/10).

**Supplemental Tables**

**Table S1:** Inclusion and exclusion criteria for patients of the Cognition.Matters-HF study.

| **Inclusion criteria** |
| --- |
| 1. Diagnosis of systolic heart failure (left ventricular ejection fraction ≤55%; any etiology or severity) or diastolic heart failure (any etiology or severity), according to the then current guidelines (13), present for at least one year up to a maximum of fifteen years |
| 1. Age ≥ 18 years |
| 1. Written informed consent |
| **Exclusion criteria** |
| 1. Previous stroke of any severity |
| 1. Carotid artery stenosis >50% |
| 1. Previous intracranial hemorrhage of any severity |
| 1. Device implanted that impedes cranial magnet resonance imaging |
| 1. Apparent psychiatric disease (especially depression or dementia) |

**Table S2:** Outcome variables of the cognitive test battery.

| **Test** | **Cognitive domain (specification)** | **Outcome variable (T-values)** |
| --- | --- | --- |
| **Test battery of Attentional Performance (TAP)** | | |
| TAP Alertness | Intensity of attention or alertness (reaction times) | Median of reaction times with and without previous acoustic signal |
| TAP GoNoGo | Selectivity of attention (focusing, response inhibition) | Number of errors |
| TAP Divided attention | Selectivity of attention (dividing) | Number of omissions |
| TAP Incompatibility | Selectivity of attention (dividing, interference control) | Number of errors, incompatible condition (specific value visual field x hand) |
| **Visual and Verbal Memory Test (VVM2)** | | |
| Map | Short- and medium-term visual memory | Number of correct crosses |
| Text | Short- and medium-term verbal memory | Number of correct responses |
| **Wechsler-Memory-Scale (WMS-R)** | | |
| Digit span | Verbal working memory | Number of correctly remembered series |
| Block tapping span | Visual working memory | Number of correctly touched series |
| **Regensburger Word Fluency Test (RWT)** | | |
| Lexically fluency | Verbal fluency (spontaneous cognitive flexibility) | Number of correct words |
| Lexically change of categories | Verbal fluency (spontaneous cognitive flexibility, shifting) | Number of correct words |
| **Hamasch-5-Point-Test (H5PT)** | | |
|  | Visual fluency (spontaneous cognitive flexibility) | Number of correct patterns |

**Table S3:** Imaging protocol, sequence parameters and specifications.

| **Sequence** | **repetition time** (ms) | **echo time**  (ms) | **slice thickness** (mm) | **in-plane resolution** (mm) | **specifications** |
| --- | --- | --- | --- | --- | --- |
| T1w FLASH^*^ | 260 | 4.67 | 3.0 | 0.7 x 0.6 | 2D GRE |
| T1w 3D TFL^*^ | 2530 | 2.15 | 1.0 | 1.0 x 1.0 | MPRAGE,  TI 1100 ms |
| T2w FLAIR^*^ | 9000 | 135 | 3.0 | 0.9 x 0.9 | TI 2440 ms |
| T2w TSE^*^ | 6100 | 100 | 3.0 | 0.7 x 0.5 | FA 120° |
| DWI | 6500 | 91 | 3.0 | 1.9 x 1.9 | b1 0 s/mm²;  b2 1000 s/mm² |
| Localizers^1^ | 250 | 2.46 | 3.0 | 1.3 x 0.9 | Triplanar |
| SV-Spectroscopy | 2000 | 135 | 20^2^ | (20 x 20) | L, R Parietal SVS |
| ASL Perfusion | 2500 | 11 | 6 | 3.0 x 3.0 | Pulsed; TI1 1800 ms; TI2 700 ms |

ASL = arterial spin labeling; DWI = diffusion-weighted imaging; FA = flip angle; FLAIR = fluid-attenuated inversion recovery; FLASH = fast low-angle shot; GRE = gradient recalled echo; L = left; MPRAGE = magnetization-prepared rapid gradient-echo; R = right; SVS = single voxel spectroscopy; TE = echo time; TFL = turbo fast low-angle shot; TI = inversion time; TR = repetition time; TSE = turbo spin echo; ^1^ for Spectroscopy planning; ^2^ Single voxel dimensions.

**Table S4:** Spearman’s correlation of biomarkers to cognitive domains and brain morphology

|  | **Neurofilament light chain (pg/ml)** | | **Phospho tau protein (pg/ml)** | |
| --- | --- | --- | --- | --- |
|  | **ρ** | **p** | **ρ** | **p** |
| Intensity of attention (T-score) | -0.13 | 0.116 | -0.08 | 0.222 |
| Visual/verbal memory (T-score) | -0.21 | **0.013** | -0.25 | **0.002** |
| Executive functions (T-score) | -0.11 | 0.197 | -0.04 | 0.661 |
| - Selectivity of attention (T-score) | -0.13 | 0.128 | -0.17 | **0.037** |
| - Working memory (T-score) | -0.08 | 0.324 | 0.04 | 0.597 |
| - Visual/verbal fluency (T-score) | -0.02 | 0.859 | 0.02 | 0.812 |
| WMH volume (mm³) | 0.41 | **<0.001** | 0.21 | **0.011** |
| Cerebral atrophy score (1-8) | 0.45 | **<0.001** | 0.33 | **<0.001** |
| Hippocampal atrophy score (0-4) | 0.18 | **0.036** | 0.28 | **0.001** |

ρ = Spearman’s rho; WMH = white matter hyperintensity

**Table S5:** Descriptive clinical characteristics of study participants according to serum neurofilament light chain (NfL) quartiles.

|  | **n** | **Total** | **< 16.7 pg/ml**  (n = 36) |  | **16.7–26.3 pg/ml**  (n = 36) |  | **26.4–41.9 pg/ml**  (n=38) |  | **≥ 42.0 pg/ml**  (n=36) | **T** | **p** | **p_(adjusted)_** |
| --- | --- | --- | --- | --- | --- | --- | --- | --- | --- | --- | --- | --- |
| Age (years) | 146 | 63.8 (10.8) | 53:5 (9.5) |  | 62.7 (9.1) |  | 67.9 (7.5) |  | 70.6 (8.7) | 8.62 | <0.001 | <0.001 |
| Female sex | 146 | 22 (15.1%) | 6 (16.7%) |  | 5 (13.9%) |  | 5 (13.2%) |  | 6 (16.7%) | - | 0.962 | 0.962 |
| Body mass index (kg/m²) | 145 | 29.1 (5.2) | 31.1 (7.5) |  | 28.6 (3.7) |  | 28.9 (4.8) |  | 27.8 (3.4) | -2.45 | 0.016 | 0.065 |
| Systolic blood pressure (mmHg) | 145 | 138.1 (19.9) | 130.8 (16.5) |  | 134.8 (20.8) |  | 142.4 (18.1) |  | 144.5 (21.5) | 3.43 | 0.001 | 0.006 |
| Diastolic blood pressure (mmHg) | 145 | 81.0 (11.0) | 82.5 (10.3) |  | 77.2 (8.2) |  | 83.1 (10.8) |  | 81.3 (13.8) | 0.28 | 0.781 | 0.834 |
| Heart rate (beats per minute) | 145 | 64.5 (10.4) | 64.3 (10.2) |  | 67.1 (10.4) |  | 62.6 (10.0) |  | 64.4 (11.2) | -0.53 | 0.593 | 0.670 |
| **Parameters of heart failure** | | | | | | | | | | | | |
| Duration of heart failure (years) | 145 | 6.31 (6.03) | 4.50 (3.90) |  | 5.06 (5.29) |  | 7.47 (7.45) |  | 8.20 (6.26) | 3.08 | 0.003 | 0.015 |
| New York Heart Association class |  |  |  |  |  |  |  |  |  |  | 0.275 | 0.418 |
| - I | 146 | 39 (26.7%) | 13 (36.1%) |  | 9 (25.0%) |  | 11 (28.9%) |  | 6 (16.7%) |  |  |  |
| - II | 146 | 88 (60.3%) | 19 (52.8%) |  | 25 (69.4%) |  | 22 (57.9%) |  | 22 (61.1%) |  |  |  |
| - III | 146 | 19 (13.0%) | 4 (11.1%) |  | 2 (5.6%) |  | 5 (13.2%) |  | 8 (22.2%) |  |  |  |
| 6-minute walking test distance (m) | 138 | 391.5 (99.3) | 426.9 (68.8) |  | 413.1 (112.6) |  | 366.7 (108.5) |  | 358.8 (87.2) | -3.44 | 0.001 | 0.006 |
| Peripheral edema | 145 | 25 (17.2%) | 4 (11.1%) |  | 5 (13.9%) |  | 6 (15.8%) |  | 10 (28.6%) |  | 0.218 | 0.401 |
| Fatigue / weakness | 145 | 58 (40.0%) | 18 (50.0%) |  | 10 (27.8%) |  | 13 (34.2%) |  | 17 (48.6%) |  | 0.148 | 0.300 |
| Ischemic heart failure | 146 | 50 (34.2%) | 13 (36.1%) |  | 15 (41.7%) |  | 9 (23.7%) |  | 13 (36.1%) |  | 0.411 | 0.551 |
| Performing regular physical activity | 146 | 117 (80.1%) | 26 (72.2%) |  | 33 (91.7%) |  | 28 (73.3%) |  | 30 (83.3%) |  | 0.130 | 0.278 |
| Guideline-recommended heart failure therapy | 146 | 123 (84.2%) | 30 (83.3%) |  | 33 (91.7%) |  | 32 (84.2%) |  | 28 (77.8%) |  | 0.449 | 0.581 |
| **Pre-existent conditions** | | | | | | | | | | | | |
| Atrial fibrillation / atrial flutter | 146 | 33 (22.6%) | 8 (22.2%) |  | 4 (11.1%) |  | 13 (34.2%) |  | 8 (22.2%) |  | 0.130 | 0.285 |
| Coronary artery disease | 146 | 100 (68.5%) | 23 (63.9%) |  | 21 (58.3%) |  | 31 (81.6%) |  | 25 (69.4%) |  | 0.164 | 0.316 |
| History of myocardial infarction | 146 | 80 (54.8%) | 21 (58.3%) |  | 18 (50.0%) |  | 25 (65.8%) |  | 16 (44.4%) |  | 0.269 | 0.417 |
| History of revascularisation | 146 | 71 (48.6%) | 17 (47.2%) |  | 16 (44.4%) |  | 22 (57.9%) |  | 16 (44.4%) |  | 0.572 | 0.664 |
| History of coronary artery bypass grafting | 146 | 36 (24.7%) | 4 (11.1%) |  | 8 (22.2%) |  | 13 (34.2%) |  | 11 (30.6%) |  | 0.102 | 0.230 |
| Peripheral artery disease | 146 | 14 (9.6%) | 2 (5.6%) |  | 3 (8.3%) |  | 4 (10.5%) |  | 5 (13.9%) |  | 0.671 | 0.737 |
| Polyneuropathy | 146 | 8 (5.5%) | 0 (0.0%) |  | 2 (5.6%) |  | 2 (5.3%) |  | 4 (11.1%) |  | 0.231 | 0.389 |
| **Cardio-vascular risk factors** | | | | | | | | | | | | |
| Diabetes mellitus type II* | 146 | 42 (28.8%) | 6 (16.7%) |  | 11 (30.6%) |  | 11 (28.9%) |  | 14 (38.9%) |  | 0.219 | 0.393 |
| Arterial hypertension† | 146 | 116 (79.5%) | 24 (66.7%) |  | 30 (83.3%) |  | 29 (76.3%) |  | 33 (91.7%) |  | 0.059 | 0.155 |
| Hyperlipidemia‡ | 146 | 105 (71.9%) | 27 (75.0%) |  | 24 (66.7%) |  | 30 (78.9%) |  | 24 (66.7%) |  | 0.556 | 0.665 |
| (Former) smoking | 146 | 88 (60.3%) | 26 (72.2%) |  | 18 (50.0%) |  | 24 (63.2%) |  | 20 (55.6%) |  | 0.241 | 0.380 |
| **Transthoracic echocardiography** | | | | | | | | | | | | |
| LV end-diastolic dimension (mm) | 146 | 60.0 (7.2) | 62.3 (6.7) |  | 59.3 (6.9) |  | 58.5 (6.8) |  | 60.0 (8.2) | -1.47 | 0.143 | 0.298 |
| LV end-systolic dimension (mm) | 141 | 46.8 (8.1) | 47.2 (8.2) |  | 45.6 (7.7) |  | 45.7 (7.5) |  | 48.7 (9.1) | 0.73 | 0.466 | 0.594 |
| Septal wall thickness, end-diastolic (mm) | 146 | 10.6 (1.9) | 10.2 (1.8) |  | 10.5 (2.1) |  | 10.7 (1.7) |  | 11.0 (1.8) | 1.84 | 0.068 | 0.168 |
| Posterior wall thickness, end-diastolic (mm) | 145 | 9.74 (1.66) | 9.6 (1.6) |  | 9.3 (1.5) |  | 9.8 (1.6) |  | 10.2 (1.9) | 1.97 | 0.051 | 0.143 |
| Left atrial end-systolic volume index (ml/m²) | 143 | 42.0 (17.5) | 36.8 (12.4) |  | 38.9 (15.7) |  | 39.4 (14.2) |  | 52.0 (21.1) | 3.77 | <0.001 | 0.003 |
| Left ventricular ejection fraction (%) | 146 | 42.5 (8.2) | 41.8 (8.4) |  | 42.7 (7.1) |  | 43.5 (9.9) |  | 42.0 (7.1) | 0.28 | 0.783 | 0.824 |
| LV volume end-diastolic (ml) | 142 | 138.4 (45.1) | 145.3 (40.3) |  | 146.7 (47.1) |  | 130.4 (47.4) |  | 131.9 (44.6) | -1.68 | 0.096 | 0.223 |
| LV volume end-systolic (ml) | 142 | 80.4 (36.0) | 85.9 (33.7) |  | 80.7 (36.0) |  | 76.9 (41.3) |  | 78.3 (32.9) | -0.96 | 0.336 | 0.466 |
| Basal right ventricular diameter (mm) | 137 | 32.2 (5.8) | 31.5 (6.2) |  | 32.4 (6.0) |  | 31.6 (5.2) |  | 33.6 (5.9) | 1.22 | 0.226 | 0.396 |
| E (cm/s) | 146 | 79.7 (26.5) | 76.8 (21.9) |  | 78.4 (29.5) |  | 78.4 (24.4) |  | 85.1 (29.6) | 1.26 | 0.209 | 0.393 |
| A (cm/s) | 119 | 73.8 (28.0) | 74.1 (29.7) |  | 71.9 (25.2) |  | 78.7 (24.6) |  | 70.2 (33.0) | -0.17 | 0.862 | 0.885 |
| E/A ratio | 119 | 1.37 (1.25) | 1.2 (0.8) |  | 1.5 (1.6) |  | 1.1 (0.8) |  | 1.7 (1.6) | 0.99 | 0.323 | 0.455 |
| Deceleration time (ms) | 146 | 191.6 (54.7) | 203.1 (59.0) |  | 198.3 (56.6) |  | 184.7 (50.4) |  | 180.9 (51.6) | -2.00 | 0.047 | 0.139 |
| e' (cm/s) | 142 | 7.98 (2.93) | 8.1 (2.5) |  | 8.4 (3.1) |  | 8.1 (3.5) |  | 7.2 (2.5) | -1.43 | 0.157 | 0.309 |
| E/e‘ ratio | 142 | 10.9 (4.3) | 10.0 (3.5) |  | 10.3 (4.9) |  | 10.9 (4.1) |  | 12.6 (4.4) | 2.60 | 0.010 | 0.051 |
| a‘ (cm/s) | 112 | 6.96 (2.82) | 7.0 (2.5) |  | 7.4 (3.5) |  | 6.5 (2.1) |  | 6.8 (2.9) | -0.17 | 0.862 | 0.673 |
| Max. systolic trans-tricuspid gradient (mmHg) | 112 | 30.5 (10.6) | 27.3 (6.7) |  | 31.5 (13.0) |  | 30.8 (9.7) |  | 31.5 (11.2) | 1.21 | 0.230 | 0.395 |
| **Laboratory results** | | | | | | | | | | | | |
| Alanine aminotransferase (U/l) | 145 | 28.1 (14.1) | 34.9 (17.9) |  | 27.2 (9.5) |  | 25.5 (12.9) |  | 25.0 (12.9) | -3.10 | 0.002 | 0.016 |
| Albumin (g/dl) | 146 | 4.55 (0.26) | 4.57 (0.29) |  | 4.59 (0.22) |  | 4.54 (0.29) |  | 4.52 (0.24) | -1.03 | 0.304 | 0.437 |
| Aspartate aminotransferase (U/l) | 144 | 27.2 (8.9) | 28.2 (13.0) |  | 26.5 (6.4) |  | 26.6 (7.0) |  | 27.4 (8.0) | -0.35 | 0.731 | 0.791 |
| Cholesterol (mg/dl) | 146 | 188.9 (56.2) | 196.9 (72.9) |  | 182.6 (40.0) |  | 186.7 (51.2) |  | 189.3 (57.3) | -0.45 | 0.656 | 0.730 |
| Creatine kinase (U/l) | 146 | 125.5 (77.8) | 129.6 (86.6) |  | 131.2 (95.2) |  | 118.0 (66.6) |  | 123.7 (60.7) | -0.54 | 0.591 | 0.677 |
| Creatine kinase, muscle-brain (U/l) | 129 | 17.1 (4.2) | 17.0 (3.9) |  | 16.3 (4.2) |  | 17.4 (3.9) |  | 17.8 (5.0) | 1.06 | 0.291 | 0.426 |
| C-reactive protein (mg/dl) | 146 | 0.43 (0.84) | 0.42 (0.97) |  | 0.38 (0.51) |  | 0.57 (1.24) |  | 0.34 (0.33) | -0.10 | 0.922 | 0.934 |
| Iron (µg/dl) | 146 | 92.8 (32.7) | 96.7 (35.3) |  | 100.5 (31.6) |  | 93.1 (34.1) |  | 81.3 (27.2) | -2.23 | 0.027 | 0.099 |
| Ferritin (µg/l) | 145 | 235 (184.5) | 264 (198) |  | 272 (149) |  | 223 (172) |  | 186 (208) | -2.10 | 0.041 | 0.128 |
| Fibrinogen (g/l) | 145 | 3.51 (0.76) | 3.32 (0.76) |  | 3.50 (0.69) |  | 3.50 (0.68) |  | 3.71 (0.91) | 2.07 | 0.040 | 0.133 |
| γ-glutamyltransferase (U/l) | 145 | 67.6 (107.2) | 59.8 (130.1) |  | 48.5 (37.8) |  | 79.7 (108.8) |  | 81.3 (125.2) | 1.20 | 0.232 | 0.382 |
| Hemoglobin (g/dl) | 145 | 14.3 (1.4) | 15.1 (1.1) |  | 14.1 (1.3) |  | 14.3 (1.2) |  | 13.8 (1.8) | -3.48 | 0.001 | 0.007 |
| Uric acid (mg/dl) | 146 | 6.60 (1.67) | 6.11 (1.30) |  | 6.45 (1.45) |  | 6.75 (1.64) |  | 7.08 (2.10) | 2.64 | 0.009 | 0.049 |
| Urea (mg/dl) | 146 | 42.6 (16.4) | 32.6 (8.7) |  | 39.8 (11.3) |  | 44.8 (18.1) |  | 53.0 (18.3) | 6.07 | <0.001 | <0.001 |
| Hemoglobin A1c (%) | 146 | 6.23 (1.19) | 5.8 (0.7) |  | 6.1 (0.9) |  | 6.5 (1.4) |  | 6.5 (1.4) | 2.90 | 0.004 | 0.024 |
| High density lipoprotein (mg/dl) | 145 | 47.8 (13.0) | 49.1 (10.9) |  | 49.1 (16.7) |  | 44.9 (11.0) |  | 48.0 (12.8) | -0.79 | 0.431 | 0.567 |
| Hematocrit (%) | 146 | 42.1 (4.2) | 43.7 (2.9) |  | 40.9 (5.5) |  | 42.4 (2.9) |  | 41.4 (4.6) | -1.74 | 0.084 | 0.202 |
| Potassium (mmol/l) | 146 | 4.51 (0.47) | 4.39 (0.43) |  | 4.51 (0.44) |  | 4.53 (0.45) |  | 4.64 (0.53) | 2.25 | 0.026 | 0.097 |
| Complement factor C3c (mg/dl) | 146 | 122.0 (18.3) | 125.8 (18.8) |  | 121.3 (16.4) |  | 124.4 (21.2) |  | 116.4 (15.1) | -1.85 | 0.066 | 0.169 |
| Mean corp. hemoglobin concentr. (g/dl) | 146 | 33.8 (1.2) | 34.4 (1.1) |  | 33.9 (1.1) |  | 33.7 (1.2) |  | 33.3 (1.1) | -4.31 | <0.001 | <0.001 |
| Mean corpuscular hemoglobin (pg) | 146 | 30.4 (1.9) | 30.5 (1.5) |  | 30.6 (1.9) |  | 30.6 (1.9) |  | 30.2 (2.2) | -0.60 | 0.554 | 0.673 |
| Mean corpuscular volume (fl) | 145 | 89.9 (4.6) | 88.5 (3.9) |  | 90.0 (4.2) |  | 90.6 (4.6) |  | 90.6 (5.3) | 2.03 | 0.045 | 0.136 |
| Natrium (mmol/l) | 146 | 139.6 (2.3) | 139.4 (2.0) |  | 138.9 (2.0) |  | 139.6 (2.4) |  | 140.6 (2.6) | 2.48 | 0.014 | 0.063 |
| N-terminal pro B natriuretic peptide (pg/ml) | 146 | 1330 (2041) | 491 (495) |  | 879 (1.083) |  | 1.317 (1.611) |  | 2.636 (3.249) | 4.69 | <0.001 | <0.001 |
| eGFR (ml/min/1.73 m²) | 140 | 66.5 (19.4) | 81.9 (14.1) |  | 68.8 (16.3) |  | 64.7 (15.5) |  | 50.6 (18.2) | -8.16 | <0.001 | <0.001 |
| Triglycerides (mg/dl) | 146 | 199 (329.8) | 260.0 (643.2) |  | 174.6 (95.1) |  | 204.7 (128.0) |  | 160.1 (67.1) | -1.10 | 0.275 | 0.410 |
| Thrombocytes (10³/µl) | 145 | 214.0 (55.1) | 237.9 (53.7) |  | 224.1 (51.1) |  | 197.4 (44.7) |  | 198.4 (61.1) | -3.68 | <0.001 | 0.004 |
| Transferrin (mg/dl) | 145 | 274.2 (46.3) | 276.5 (54.1) |  | 266.5 (37.8) |  | 266.3 (36.3) |  | 287.8 (52.9) | 0.96 | 0.341 | 0.464 |
| Transferrin saturation (%) | 145 | 27.7 (9.4) | 25.6 (10.4) |  | 27.4 (9.9) |  | 25.0 (8.9) |  | 20.9 (7.7) | -2.37 | 0.019 | 0.076 |
| Thyroid-stimulating hormone (mIU/l) | 127 | 1.61 (1.65) | 1.3 (0.9) |  | 1.3 (0.9) |  | 1.6 (1.0) |  | 2.2 (2.9) | 2.16 | 0.033 | 0.113 |
| **Medication** | | | | | | | | | | | | |
| Angiotensin-converting enzyme inhibitor | 146 | 86 (58.9%) | 23 (63.9%) |  | 19 (52.8%) |  | 23 (60.5%) |  | 21 (58.3%) |  | 0.808 | 0.839 |
| Beta-blocker | 146 | 131 (89.7%) | 30 (83.3%) |  | 33 (91.7%) |  | 35 (92.1%) |  | 33 (91.7%) |  | 0.547 | 0.675 |
| Aldosteron antagonist | 146 | 54 (37.0%) | 12 (33.3%) |  | 19 (52.8%) |  | 15 (39.5%) |  | 8 (22.3%) |  | 0.057 | 0.155 |
| Diuretics | 146 | 80 (54.8%) | 11 (30.6%) |  | 18 (50.0%) |  | 23 (60.5%) |  | 28 (77.8%) |  | 0.001 | 0.006 |
| Acetylsalicylic acid | 146 | 80 (54.8%) | 22 (61.1%) |  | 19 (52.8%) |  | 24 (63.2%) |  | 15 (41.7%) |  | 0.239 | 0.385 |
| Other platelet aggregation inhibitors | 146 | 21 (14.4%) | 4 (11.1%) |  | 8 (22.2%) |  | 5 (13.2%) |  | 4 (11.1%) |  | 0.481 | 0.603 |
| Coumadin or novel oral anticoagulant | 146 | 45 (30.8%) | 6 (16.7%) |  | 7 (19.4%) |  | 14 (36.8%) |  | 17 (47.2%) |  | 0.013 | 0.058 |

Metric data are displayed as mean (standard deviation). For the detection of trends, regression with quartiles as independent variable was applied. Chi-square test was used to test for differences between expected and observed frequencies; if the expected value was below five, Fisher’s exact test was used.

6-MWT = 6-minute walk test; E = Early diastolic mitral valve inflow velocity, e´= Eearly diastolic mitral annular relaxation velocity, eGFR = Estimated glomerular filtration rate according to MDRD formula; LA = Left atrium; LV = Left ventricle; n = Valid values; NfL = Neurofilament light chain; NYHA = New York Heart association; *p* = two-sided p-value; p_(adjusted)_ = Adjusted p-value after false discovery rate approach using the Benjamini–Hochberg procedure; * = History of diabetes mellitus type II or HbA1c >6.5%; † = Sitting blood pressure >140/90 mmHg or history of hypertension before onset of heart failure; ‡ = Hyperlipidemia or statin treatment.**Table S6:** Descriptive clinical characteristics of study participants according to serum phosphorylated tau protein (pTau) quartiles.

|  | **n** | **Total** | **< 1.09 pg/ml**  (n = 36) |  | **1.09-1.56 pg/ml**  (n = 37) |  | **1.57-2.40 pg/ml**  (n=37) |  | **≥ 2.41 pg/ml**  (n=36) | **T** | | **p** | **p_(adjusted)_** |
| --- | --- | --- | --- | --- | --- | --- | --- | --- | --- | --- | --- | --- | --- |
| Age (years) | 146 | 63.8 (10.8) | 56.1 (10.5) |  | 63.0 (9.4) |  | 66.3 (10.6) |  | 69.6 (8.3) | 6.07 | | <0.001 | <0.001 |
| Female sex | 146 | 22 (15.1%) | 7 (19.4%) |  | 6 (16.2%) |  | 6 (16.2%) |  | 3 (8.3%) |  | | 0.595 | 0.771 |
| Body mass index (kg/m²) | 145 | 29.1 (5.2) | 30.3 (7.7) |  | 29.1 (4.0) |  | 28.3 (4.4) |  | 28.9 (4.0) | -1.31 | | 0.192 | 0.434 |
| Systolic blood pressure (mmHg) | 145 | 138.1 (19.9) | 134.4 (15.9) |  | 137.7 (18.6) |  | 138.5 (22.7) |  | 141.9 (21.6) | 1.58 | | 0.117 | 0.307 |
| Diastolic blood pressure (mmHg) | 145 | 81.0 (11.0) | 83.0 (8.6) |  | 79.9 (11.5) |  | 81.5 (15.0) |  | 79.9 (7.7) | -0.90 | | 0.372 | 0.600 |
| Heart rate (beats per minute) | 145 | 64.5 (10.4) | 63.5 (8.8) |  | 67.2 (10.6) |  | 65.9 (12.2) |  | 61.5 (9.3) | -0.95 | | 0.343 | 0.589 |
| **Parameters of heart failure** | | | | | | | | | | | | | |
| Duration of heart failure (years) | 145 | 6.31 (6.03) | 4.50 (4.90) |  | 4.42 (5.00) |  | 7.24 (6.06) |  | 9.06 (6.89) | 3.84 | | <0.001 | 0.002 |
| New York Heart Association class |  |  |  |  |  |  |  |  |  |  | | 0.424 | 0.632 |
| I | 146 | 39 (26.7%) | 11 (30.6%) |  | 14 (37.8%) |  | 7 (18.9%) |  | 7 (19.4%) |  | |  |  |
| II | 146 | 88 (60.3%) | 21 (58.3%) |  | 20 (54.1%) |  | 25 (67.6%) |  | 22 (61.1%) |  | |  |  |
| III | 146 | 19 (13.0%) | 4 (11.1%) |  | 3 (8.1%) |  | 5 (13.5%) |  | 7 (19.4) |  | |  |  |
| 6-minute walking test distance (m) | 138 | 391.5 (99.3) | 415.0 (90.9) |  | 400.0 (105.7) |  | 393.0 (103.4) |  | 357.7 (91.3) | -2.395 | | 0.018 | 0.079 |
| Peripheral edema | 145 | 25 (17.2%) | 5 (13.9%) |  | 5 (13.5%) |  | 5 (13.5%) |  | 10 (28.6%) |  | | 0.245 | 0.510 |
| Fatigue / weakness | 145 | 58 (40.0%) | 17 (47.2%) |  | 16 (43.2%) |  | 14 (37.8%) |  | 11 (31.4%) |  | | 0.554 | 0.755 |
| Ischemic heart failure | 146 | 50 (34.2%) | 14 (38.9%) |  | 14 (37.8%) |  | 13 (35.1%) |  | 9 (25.0%) |  | | 0.586 | 0.784 |
| Performing regular physical activity | 146 | 117 (80.1%) | 27 (75.0%) |  | 31 (83.8%) |  | 29 (78.4%) |  | 30 (83.3%) |  | | 0.751 | 0.872 |
| Guideline-recommended heart failure therapy | 146 | 123 (84.2%) | 32 (88.9%) |  | 31 (83.8%) |  | 32 (86.5%) |  | 28 (77.8%) |  | | 0.601 | 0.766 |
| **Pre-existent conditions** | | | | | | | | | | | | | |
| Atrial fibrillation / atrial flutter | 146 | 33 (22.6%) | 4 (11.1%) |  | 6 (16.2%) |  | 10 (27.0%) |  | 13 (36.1%) |  | | 0.051 | 0.185 |
| Coronary artery disease | 146 | 100 (68.5%) | 22 (61.1%) |  | 23 (62.2%) |  | 26 (70.3%) |  | 29 (80.6%) |  | | 0.253 | 0.513 |
| History of myocardial infarction | 146 | 80 (54.8%) | 20 (55.6%) |  | 20 (54.1%) |  | 18 (48.6%) |  | 22 (61.1%) |  | | 0.762 | 0.873 |
| History of revascularisation | 146 | 71 (48.6%) | 15 (41.7%) |  | 19 (51.4%) |  | 20 (54.1%) |  | 17 (47.2%) |  | | 0.626 | 0.773 |
| History of coronary artery bypass grafting | 146 | 36 (24.7%) | 6 (16.7%) |  | 9 (24.3%) |  | 12 (32.4%) |  | 9 (25.0%) |  | | 0.485 | 0.684 |
| Peripheral artery disease | 146 | 14 (9.6%) | 4 (11.1%) |  | 3 (8.1%) |  | 3 (8.1%) |  | 4 (11.1%) |  | | 0.944 | >0.999 |
| Polyneuropathy | 146 | 8 (5.5%) | 0 (0.0%) |  | 3 (8.1%) |  | 0 (0.0%) |  | 5 (13.9%) |  | | 0.022 | 0.091 |
| **Cardio-vascular risk factors** | | | | | | | | | | | | | |
| Diabetes mellitus type II* | 146 | 42 (28.8%) | 9 (25.0%) |  | 13 (35.1%) |  | 7 (18.9%) |  | 13 (36.1%) |  | | 0.298 | 0.535 |
| Arterial hypertension^†^ | 146 | 116 (79.5%) | 26 (72.2%) |  | 27 (73.0%) |  | 31 (83.8%) |  | 32 (88.9%) |  | | 0.213 | 0.467 |
| Hyperlipidemia‡ | 146 | 105 (71.9%) | 26 (72.2%) |  | 24 (64.9%) |  | 27 (73.0%) |  | 28 (77.8%) |  | | 0.672 | 0.817 |
| (Former) smoking | 146 | 88 (60.3%) | 24 (66.7%) |  | 22 (59.5%) |  | 22 (59.5%) |  | 20 (55.6%) |  | | 0.809 | 0.913 |
| **Transthoracic echocardiography** | | | | | | | | | | | | | |
| LV end-diastolic dimension (mm) | 146 | 60.0 (7.2) | 59.4 (5.6) |  | 59.5 (5.4) |  | 60.4 (9.2) |  | 60.8 (8.2) | 0.95 | | 0.343 | 0.577 |
| LV end-systolic dimension (mm) | 141 | 46.8 (8.1) | 45.4 (6.4) |  | 45.6 (7.1) |  | 47.6 (10.1) |  | 48.7 (8.4) | 1.94 | | 0.054 | 0.186 |
| Septal wall thickness. end-diastolic (mm) | 146 | 10.6 (1.9) | 10.4 (1.9) |  | 10.5 (2.3) |  | 10.8 (1.8) |  | 10.7 (1.5) | 0.73 | | 0.464 | 0.667 |
| Posterior wall thickness. end-diastolic (mm) | 145 | 9.74 (1.66) | 9.6 (1.7) |  | 9.5 (1.8) |  | 9.9 (1.6) |  | 9.9 (1.5) | 1.12 | | 0.265 | 0.510 |
| Left atrial end-systolic volume index (ml/m²) | 143 | 42.0 (17.5) | 34.5 (12.4) |  | 38.5 (13.7) |  | 42.9 (19.2) |  | 51.1 (18.1) | 4.51 | | <0.001 | <0.001 |
| Left ventricular ejection fraction (%) | 146 | 42.5 (8.2) | 43.8 (7.3) |  | 43.1 (6.8) |  | 41.9 (10.4) |  | 41.1 (7.8) | -1.49 | | 0.139 | 0.343 |
| LV volume end-diastolic (ml) | 142 | 138.4 (45.1) | 132.1 (38.9) |  | 135.0 (38.2) |  | 143.7 (55.4) |  | 142.3 (46.5) | 1.14 | | 0.255 | 0.504 |
| LV volume end-systolic (ml) | 142 | 80.4 (36.0) | 70.1 (21.6) |  | 76.6 (25.8) |  | 86.7 (48.3) |  | 87.4 (39.6) | 2.318 | | 0.022 | 0.086 |
| Basal right ventricular diameter (mm) | 137 | 32.2 (5.8) | 30.5 (4.9) |  | 31.8 (5.1) |  | 32.3 (6.3) |  | 34.3 (6.4) | 2.753 | | 0.007 | 0.038 |
| E (cm/s) | 146 | 79.7 (26.5) | 75.0 (23.5) |  | 81.1 (25.7) |  | 79.9 (30.1) |  | 82.5 (26.4) | 1.07 | | 0.285 | 0.523 |
| A (cm/s) | 119 | 73.8 (28.0) | 77.3 (21.6) |  | 71.0 (32.3) |  | 74.6 (27.4) |  | 71.6 (31.3) | -0.60 | | 0.552 | 0.765 |
| E/A ratio | 119 | 1.37 (1.25) | 1.11 (0.74) |  | 1.47 (1.07) |  | 1.32 (1.48) |  | 1.65 (1.67) | 1.41 | | 0.162 | 0.389 |
| Deceleration time (ms) | 146 | 191.6 (54.7) | 190.8 (52.3) |  | 190.1 (56.3) |  | 198.1 (60.4) |  | 187.4 (50.8) | -0.05 | | 0.963 | >0.999 |
| e' (cm/s) | 142 | 7.98 (2.93) | 7.69 (2.56) |  | 8.58 (3.28) |  | 7.62 (2.91) |  | 8.03 (2.93) | 0.02 | | 0.988 | 0.988 |
| E/e‘ ratio | 142 | 10.9 (4.3) | 10.6 (3.8) |  | 10.7 (4.9) |  | 11.6 (5.4) |  | 10.8 (2.8) | 0.51 | | 0.610 | 0.765 |
| a‘ (cm/s) | 112 | 6.96 (2.82) | 7.59 (2.83) |  | 6.70 (2.55) |  | 6.41 (3.03) |  | 7.04 (2.93) | -0.95 | | 0.347 | 0.570 |
| Max. systolic trans-tricuspid gradient (mmHg) | 112 | 30.5 (10.6) | 29.8 (13.9) |  | 28.7 (6.7) |  | 30.1 (8.9) |  | 32.8 (11.6) | 1.20 | | 0.231 | 0.494 |
| **Laboratory findings** | | | | | | | | | | | | | |
| Alanine aminotransferase (U/l) | 145 | 28.1 (14.1) | 32.2 (18.1) |  | 29.9 (10.4) |  | 26.1 (12.4) |  | 24.4 (13.6) | | -2.65 | 0.009 | 0.042 |
| Albumin (g/dl) | 146 | 4.55 (0.26) | 4.6 (0.3) |  | 4.5 (0.3) |  | 4.5 (0.2) |  | 4.5 (0.2) | | -1.65 | 0.100 | 0.293 |
| Aspartate aminotransferase (U/l) | 144 | 27.2 (8.9) | 26.6 (13.1) |  | 27.1 (6.4) |  | 26.3 (5.9) |  | 28.7 (8.5) | | 0.83 | 0.409 | 0.621 |
| Cholesterol (mg/dl) | 146 | 188.9 (56.2) | 202.8 (73.5) |  | 181.7 (44.1) |  | 179.4 (41.5) |  | 191.9 (59.6) | | -0.84 | 0.402 | 0.623 |
| Creatine kinase (U/l) | 146 | 125.5 (77.8) | 95.3 (45.9) |  | 105.3 (61.9) |  | 140.1 (85.6) |  | 161.6 (92.3) | | 4.28 | <0.001 | 0.001 |
| Creatine kinase. muscle-brain (U/l) | 129 | 17.1 (4.2) | 16.5 (4.0) |  | 15.4 (2.6) |  | 17.0 (3.9) |  | 19.4 (5.2) | | 3.33 | 0.001 | 0.008 |
| C-reactive protein (mg/dl) | 146 | 0.43 (0.84) | 0.30 (0.33) |  | 0.60 (1.50) |  | 0.47 (0.55) |  | 0.33 (0.41) | | -0.04 | 0.967 | 0.993 |
| Iron (µg/dl) | 146 | 92.8 (32.7) | 95.1 (32.0) |  | 93.5 (30.4) |  | 86.4 (32.4) |  | 96.5 (36.1) | | -0.13 | 0.901 | 0.989 |
| Ferritin (µg/l) | 145 | 235 (184.5) | 317.4 (192.8) |  | 211.0 (160.3) |  | 228.5 (221.2) |  | 186.3 (129.5) | | -2.80 | 0.006 | 0.036 |
| Fibrinogen (g/l) | 145 | 3.51 (0.76) | 3.41 (0.85) |  | 3.33 (0.62) |  | 3.71 (0.79) |  | 3.59 (0.78) | | 1.62 | 0.107 | 0.292 |
| γ-glutamyltransferase (U/l) | 145 | 67.6 (107.2) | 59.3 (128.6) |  | 67.8 (73.9) |  | 48.3 (35.8) |  | 95.7 (150.7) | | 1.12 | 0.266 | 0.501 |
| Hemoglobin (g/dl) | 145 | 14.3 (1.4) | 14.6 (1.5) |  | 14.4 (1.1) |  | 13.9 (1.5) |  | 14.4 (1.5) | | -1.31 | 0.192 | 0.447 |
| Uric acid (mg/dl) | 146 | 6.60 (1.67) | 5.77 (1.37) |  | 6.48 (1.33) |  | 7.14 (1.91) |  | 6.99 (1.71) | | 3.62 | <0.001 | 0.004 |
| Urea (mg/dl) | 146 | 42.6 (16.4) | 33.5 (8.2) |  | 37.4 (9.1) |  | 46.8 (15.1) |  | 52.6 (22.1) | | 6.15 | <0.001 | <0.001 |
| Hemoglobin A1c (%) | 146 | 6.23 (1.19) | 5.90 (1.10) |  | 6.27 (1.00) |  | 6.28 (1.28) |  | 6.48 (1.32) | | 1.98 | 0.049 | 0.186 |
| High density lipoprotein (mg/dl) | 145 | 47.8 (13.0) | 47.8 (13.3) |  | 47.5 (13.3) |  | 46.2 (13.2) |  | 49.5 (12.6) | | 0.39 | 0.697 | 0.834 |
| Hematocrit (%) | 146 | 42.1 (4.2) | 41.94 (5.79) |  | 42.42 (3.06) |  | 41.39 (3.78) |  | 42.67 (3.87) | | 0.36 | 0.722 | 0.852 |
| Potassium (mmol/l) | 146 | 4.51 (0.47) | 4.48 (0.46) |  | 4.51 (0.38) |  | 4.50 (0.52) |  | 4.57 (0.51) | | 0.76 | 0.451 | 0.660 |
| Complement factor C3c (mg/dl) | 146 | 122.0 (18.3) | 125.7 (15.4) |  | 121.6 (19.0) |  | 122.8 (19.9) |  | 117.9 (18.3) | | -1.36 | 0.102 | 0.288 |
| Mean corp. hemoglobin concentr. (g/dl) | 146 | 33.8 (1.2) | 34.3 (1.4) |  | 34.0 (0.7) |  | 33.5 (1.2) |  | 33.6 (1.2) | | -2.73 | 0.007 | 0.035 |
| Mean corpuscular hemoglobin (pg) | 146 | 30.4 (1.9) | 30.4 (2.1) |  | 30.7 (1.4) |  | 30.1 (2.2) |  | 30.6 (1.8) | | -0.06 | 0.956 | >0.999 |
| Mean corpuscular volume (fl) | 145 | 89.9 (4.6) | 88.7 (4.7) |  | 90.4 (3.4) |  | 89.8 (4.9) |  | 90.9 (5.0) | | 1.83 | 0.070 | 0.221 |
| Natrium (mmol/l) | 146 | 139.6 (2.3) | 139.4 (2.2) |  | 139.6 (2.1) |  | 139.4 (2.8) |  | 139.8 (2.3) | | 0.54 | 0.589 | 0.776 |
| N-terminal pro B natriuretic peptide (pg/ml) | 146 | 1330 (2041) | 626 (1.085) |  | 723 (789) |  | 2.091 (3.134) |  | 1.884 (1.954) | | 3.47 | 0.001 | 0.006 |
| eGFR (ml/min/1.73 m²) | 140 | 66.5 (19.4) | 79.4 (15.8) |  | 72.9 (14.2) |  | 58.2 (15.2) |  | 55.5 (21.3) | | -6.87 | <0.001 | <0.001 |
| Triglycerides (mg/dl) | 146 | 199 (329.8) | 286.6 (643.1) |  | 177.1 (108.6) |  | 177.2 (85.4) |  | 159.9 (83.2) | | -1.55 | 0.123 | 0.314 |
| Thrombocytes (10³/µl) | 145 | 214.0 (55.1) | 236.8 (51.7) |  | 225.8 (49.8) |  | 205.5 (58.0) |  | 188.3 (49.5) | | -4.27 | <0.001 | <0.001 |
| Transferrin (mg/dl) | 145 | 274.2 (46.3) | 271.8 (56.0) |  | 266.5 (36.2) |  | 280.3 (56.4) |  | 278.2 (31.4) | | 0.97 | 0.335 | 0.588 |
| Transferrin saturation (%) | 145 | 27.7 (9.4) | 25.8 (10.2) |  | 25.4 (9.3) |  | 23.1 (10.0) |  | 24.6 (8.4) | | -0.85 | 0.400 | 0.632 |
| Thyroid-stimulating hormone (mIU/l) | 127 | 1.61 (1.65) | 1.2 (0.9) |  | 1.2 (0.9) |  | 2.3 (2.8) |  | 1.6 (0.8) | | 1.70 | 0.091 | 0.042 |
| **Medication** | | | | | | | | | | | | | |
| Angiotensin-converting enzyme inhibitor | 146 | 86 (58.9%) | 28 (77.8%) |  | 15 (40.5%) |  | 18 (48.6%) |  | 25 (69.4%) |  | | 0.003 | 0.022 |
| Beta-blocker | 146 | 131 (89.7%) | 32 (88.9%) |  | 33 (89.2%) |  | 33 (89.2%) |  | 33 (91.7%) |  | | 0.978 | 0.991 |
| Aldosteron antagonist | 146 | 54 (37.0%) | 14 (38.9%) |  | 13 (35.1%) |  | 15 (40.5%) |  | 12 (33.3%) |  | | 0.915 | 0.990 |
| Diuretics | 146 | 80 (54.8%) | 13 (36.1%) |  | 17 (45.9%) |  | 24 (64.9%) |  | 26 (72.2%) |  | | 0.007 | 0.036 |
| Acetylsalicylic acid | 146 | 80 (54.8%) | 26 (72.2%) |  | 21 (56.8%) |  | 18 (48.6%) |  | 15 (41.7%) |  | | 0.057 | 0.186 |
| Other platelet aggregation inhibitors | 146 | 21 (14.4%) | 6 (16.7%) |  | 4 (10.8%) |  | 5 (13.5%) |  | 6 (16.7%) |  | | 0.871 | 0.969 |
| Coumadin or novel oral anticoagulant | 146 | 45 (30.8%) | 4 (11.1%) |  | 7 (18.9%) |  | 15 (40.5%) |  | 18 (50.0%) |  | | 0.001 | 0.006 |

Metric data are displayed as mean (standard deviation). For the detection of trends, regression with quartiles as independent variable was applied. Chi-square test was used to test for differences between expected and observed frequencies; if the expected value was below five, Fisher’s exact test was used.

6-MWT = 6-minute walk test; E = early diastolic mitral valve inflow velocity. e´= early diastolic mitral annular relaxation velocity. GFR = glomerular filtration rate (MDRD formula); LA = left atrium; LV = left ventricle; n = valid values; NfL = neurofilament light chain; NYHA = New York Heart association; *p* = two-sided p-value; p_(adjusted)_ = adjusted p-value after false discovery rate approach using Benjamini–Hochberg procedure; pTau = phosphorylated Tau protein-181; * = History of diabetes mellitus type II or HbA1c >6.5%; † = Sitting blood pressure >140/90 mmHg or history of hypertension before onset of heart failure; ‡ = Hyperlipidemia or statin treatment.

**Table S7:** Mediation analysis of parameters affecting serum levels of Ln(NfL).

| Potential mediators | Effect on mediator | | Effect of mediator  on Ln(NfL) | | Indirect effect  on Ln(NfL) | | Mediation? |
| --- | --- | --- | --- | --- | --- | --- | --- |
|  | a | p | b | p |  | ab |  |
| **Age → Ln(NfL)** (c=0.554; p<0.001; c’= 0.393; p<0.001) | | | | | | | |
| NT-proBNP (pg/ml) | 0.190 | 0.003 | 0.209 | 0.046 | 0.040 | | Partial |
| eGFR (ml/min/1.73m²) | -0.401 | <0.001 | -0.285 | <0.001 | 0.114 | | Partial |
| **eGFR → Ln(NfL)** (c=-0.537; p<0.001; c’=-0.285; p<0.001) | | | | | | | |
| Age (years) | -0.401 | <0.001 | 0.393 | <0.001 | -0.158 | | Partial |
| NT-proBNP (pg/ml) | -0.391 | 0.004 | 0.209 | 0.046 | -0.082 | | Partial |
| **NT-proBNP** **→ Ln(NfL)** (c=0.395; p=0.029; c’=0.209; p=0.046) | | | | | | | |
| Age (years) | 0.189 | 0.339 | 0.393 | <0.001 | 0.074 | | Partial |
| eGFR (ml/min/1.73m²) | -0.391 | <0.001 | -0.285 | <0.001 | 0.111 | | Partial |

Standardized effect sizes are shown. a = effect of independent variable on mediator; ab = indirect effect on Ln(NfL); b = effect of mediator on Ln(NfL); c = total effect on Ln(NfL); c’ = direct effect on Ln(NfL); eGFR = estimated glomerular filtration rate

**Table S8:** Mediation analysis of parameters affecting serum levels of Ln(pTau).

| Potential mediators | Effect on mediator | | Effect of mediator  on Ln(pTau) | | Indirect effect  on Ln(pTau) | | Mediation? |
| --- | --- | --- | --- | --- | --- | --- | --- |
|  | a | p | b | p |  | ab |  |
| **Age → Ln(pTau)** (c=0.488; p<0.001; c’= 0.316; p<0.001) | | | | | | | |
| eGFR (ml/min/1.73m²) | -0.412 | <0.001 | -0.218 | 0.003 | 0.090 | | Partial |
| LAVI (ml/m²) | 0.216 | 0.006 | 0.281 | <0.001 | 0.061 | | Partial |
| Creatine kinase (U/l) | 0.044 | 0.541 | 0.375 | <0.001 |  | | - |
| Ferritin (µg/l) | -0.026 | 0.723 | -0.156 | 0.031 |  | | - |
| **eGFR → Ln(pTau)** (c=-0.490; p<0.001; c’=-0.218; p=0.001) | | | | | | | |
| Age (years) | -0.412 | <0.001 | 0.316 | <0.001 | -0.130 | | Partial |
| LAVI (ml/m²) | -0.225 | 0.019 | 0.281 | <0.001 | -0.063 | | Partial |
| Creatine kinase (U/l) | -0.121 | 0.169 | 0.375 | <0.001 |  | | - |
| Ferritin (µg/l) | 0.211 | 0.006 | -0.156 | 0.031 | -0.033 | | Partial |
| **LAVI** **→ Ln(pTau)** (c=0.408; p<0.001; c’=0.281; p=0.001) | | | | | | | |
| Age (years) | 0.216 | 0.020 | 0.316 | <0.001 | 0.068 | | Partial |
| eGFR (ml/min/1.73m²) | -0.225 | 0.005 | -0.218 | 0.003 | 0.049 | | Partial |
| Creatine kinase (U/l) | -0.016 | 0.851 | 0.375 | <0.001 |  | | - |
| Ferritin (µg/l) | -0.102 | 0.261 | -0.156 | 0.031 |  | | - |
| **Creatine kinase → Ln(pTau)** (c=0.409; p<0.001; c’=0.375; p<0.001) | | | | | | | |
| Age (years) | 0.044 | 0.564 | 0.316 | <0.001 |  | | - |
| eGFR (ml/min/1.73m²) | -0.121 | 0.120 | -0.218 | 0.003 |  | |  |
| LAVI (ml/m²) | -0.016 | 0.855 | 0.281 | <0.001 |  | | - |
| Ferritin (µg/l) | 0.012 | 0.884 | -0.156 | 0.031 |  | | - |
| **Ferritin → Ln(pTau)** (c=-0.234; p<0.001; c’=0.156; p<0.001) | | | | | | | |
| Age (years) | -0.026 | 0.727 | 0.375 | <0.001 |  | |  |
| eGFR (ml/min/1.73m²) | -0.211 | 0.047 | -0.218 | 0.003 | -0.046 | | Partial |
| LAVI (ml/m²) | 0.102 | 0.343 | 0.281 | <0.001 |  | |  |
| Creatine kinase (U/l) | 0.012 | 0.881 | 0.375 | <0.001 |  | |  |

Standardized effect sizes are shown. a = effect of independent variable on mediator; ab = indirect effect on Ln(pTau); b = effect of mediator on Ln(pTau); c = total effect on Ln(pTau); c’ = direct effect on Ln(pTau); eGFR = estimated glomerular filtration rate; LAVI = left atrial volume index

|  |  | Separate models  Univariable (enter) | | | Separate models  Multivariable (backward) | | | Combined model  Multivariable (backward) | | |
| --- | --- | --- | --- | --- | --- | --- | --- | --- | --- | --- |
|  | n | T | *p* | R² | VIF | T | p | VIF | T | *p* |
| **Serum biomarker** |  |  |  |  | R² = 0.06 | | | R² = 0.06 | | |
| Ln (Neurofilament light chain (pg/ml)) | 146 | -3.12 | 0.002 | 0.06 | 1.00 | -3.12 | 0.002 | 1.00 | -3.12 | 0.002 |
| **Clinical correlates of NfL** |  |  |  |  | R² = 0.06 | | |  | | |
| Estimated GFR (ml/min/1.73m²) | 146 | 3.03 | 0.003 | 0.06 | 1.00 | 3.03 | 0.003 |  |  |  |
| Urea (mg/dl) | 146 | -2.29 | 0.023 | 0.04 |  |  |  |  |  |  |
| NT-proBNP (pg/ml) | 140 | -2.68 | 0.008 | 0.05 |  |  |  |  |  |  |
| Mean corpuscular hemoglobin concentration (g/dl) | 146 | 2.38 | 0.018 | 0.04 |  |  |  |  |  |  |
| Left-atrial volume index (ml/m²) | 143 | -1.33 | 0.185 | 0.01 |  |  |  |  |  |  |
| Thrombocytes (10³/µl) | 145 | 0.85 | 0.399 | 0.01 |  |  |  |  |  |  |
| Systolic blood pressure (mmHg) | 145 | -1.04 | 0.299 | 0.01 |  |  |  |  |  |  |
| 6-minute walking test distance (m) | 138 | 2.24 | 0.027 | 0.03 |  |  |  |  |  |  |
| Hemoglobin (g/dl) | 146 | 2.29 | 0.023 | 0.04 |  |  |  |  |  |  |
| Duration of heart failure (years) | 145 | -0.96 | 0.340 | 0.01 |  |  |  |  |  |  |
| Alanine aminotransferase (U/l) | 145 | 2.41 | 0.017 | 0.04 |  |  |  |  |  |  |
| Hemoglobin A1c (%) | 145 | 0.21 | 0.834 | 0.00 |  |  |  |  |  |  |
| Uric acid (mg/dl) | 146 | -1.22 | 0.223 | 0.01 |  |  |  |  |  |  |

**Supplemental Table S9:** Regression analysis of age-adjusted visual/verbal memory T-score with NfL.

GFR = glomerular filtration rate (MDRD formula); *p* = two-sided p-value; R² = coefficient of determination; VIF = variance inflation factor. The T-value indicates the direction of association and the relative weight of a variable in a model; R² indicates the variance explained by the model.

**Supplemental Table S10:** Regression analysis of age-adjusted visual/verbal memory T-score with pTau.

|  |  | Separate models  Univariable (enter) | | | Separate models  Multivariable (backward) | | | Combined model  Multivariable (backward) | | |
| --- | --- | --- | --- | --- | --- | --- | --- | --- | --- | --- |
|  | n | T | *p* | R² | VIF | T | p | VIF | T | *p* |
| **Serum biomarkers** |  |  |  |  | R² = 0.06 | | | R² = 0.06 | | |
| Ln (phosphorylated Tau protein (pg/ml)) | 146 | -3.13 | 0.002 | 0.06 | 1.00 | -3.13 | 0.002 | 1.00 | -3.13 | 0.002 |
| **Clinical correlates of pTau** |  |  |  |  | R² = 0.06 | | |  | | |
| Estimated GFR (ml/min/1.73m²) | 146 | 3.03 | 0.003 | 0.06 | 1.00 | 3.03 | 0.003 |  |  |  |
| Urea (mg/dl) | 146 | -2.29 | 0.023 | 0.04 |  |  |  |  |  |  |
| Left-atrial volume index (ml/m²) | 143 | -1.33 | 0.185 | 0.01 |  |  |  |  |  |  |
| Thrombocytes (10³/µl) | 145 | 0.85 | 0.399 | 0.01 |  |  |  |  |  |  |
| Creatine kinase (U/l) | 146 | -0.15 | 0.885 | 0.00 |  |  |  |  |  |  |
| Duration of heart failure (years) | 145 | -0.96 | 0.340 | 0.01 |  |  |  |  |  |  |
| Uric acid (mg/dl) | 146 | -1.22 | 0.223 | 0.01 |  |  |  |  |  |  |
| NT-proBNP (pg/ml) | 140 | -2.68 | 0.008 | 0.05 |  |  |  |  |  |  |
| Creatine kinase, muscle-brain (U/l) | 146 | 0.02 | 0.989 | 0.00 |  |  |  |  |  |  |
| Mean corpuscular hemoglobin concentration (g/dl) | 146 | 2.38 | 0.018 | 0.04 |  |  |  |  |  |  |
| Ferritin (µg/l) | 146 | 1.30 | 0.196 | 0.01 |  |  |  |  |  |  |
| Basal right ventricular diameter (mm) | 146 | -2.03 | 0.044 | 0.03 |  |  |  |  |  |  |
| Alanine aminotransferase (U/l) | 145 | 2.41 | 0.017 | 0.04 |  |  |  |  |  |  |

GFR = glomerular filtration rate (MDRD formula); *p* = two-sided p-value; R² = coefficient of determination; VIF = variance inflation factor. The T-value indicates the direction of association and the relative weight of a variable in a model; R² indicates the variance explained by the model.

|  |  | Separate models  Univariable (enter) | | | Separate models  Multivariable (backward) | | | Combined model  Multivariable (backward) | | |
| --- | --- | --- | --- | --- | --- | --- | --- | --- | --- | --- |
|  | n | T | *p* | R² | VIF | T | p | VIF | T | *p* |
| **Serum biomarkers** |  |  |  |  | R² = 0.03 | | | R² = 0.06 | | |
| Ln (phosphorylated Tau protein (pg/ml)) | 146 | -2.13 | 0.035 | 0.03 | 1.00 | -2.13 | 0.035 | 1.00 | -2.13 | 0.035 |
| **Clinical correlates of pTau** |  |  |  |  | R² = 0.03 | | |  | | |
| Estimated GFR (ml/min/1.73m²) | 146 | 1.11 | 0.271 | 0.01 |  |  |  |  |  |  |
| Urea (mg/dl) | 146 | -1.01 | 0.315 | 0.01 |  |  |  |  |  |  |
| Left-atrial volume index (ml/m²) | 143 | -1.36 | 0.177 | 0.01 |  |  |  |  |  |  |
| Thrombocytes (10³/µl) | 145 | 1.75 | 0.082 | 0.02 |  |  |  |  |  |  |
| Creatine kinase (U/l) | 146 | -1.44 | 0.153 | 0.01 |  |  |  |  |  |  |
| Duration of heart failure (years) | 145 | -1.46 | 0.147 | 0.02 |  |  |  |  |  |  |
| Uric acid (mg/dl) | 146 | -1.12 | 0.266 | 0.01 |  |  |  |  |  |  |
| NT-proBNP (pg/ml) | 140 | -0.13 | 0.901 | 0.00 |  |  |  |  |  |  |
| Creatine kinase, muscle-brain (U/l) | 146 | 0.68 | 0.496 | 0.00 |  |  |  |  |  |  |
| Mean corpuscular hemoglobin concentration (g/dl) | 146 | -0.45 | 0.650 | 0.00 |  |  |  |  |  |  |
| Ferritin (µg/l) | 146 | 0.78 | 0.440 | 0.00 |  |  |  |  |  |  |
| Basal right ventricular diameter (mm) | 146 | -1.47 | 0.144 | 0.02 |  |  |  |  |  |  |
| Alanine aminotransferase (U/l) | 145 | 1.99 | 0.048 | 0.03 | 1.00 | 1.99 | 0.048 |  |  |  |

**Supplemental Table S11:** Regression analysis of age-adjusted selectivity of attention T-score with pTau.

GFR = glomerular filtration rate (MDRD formula); *p* = two-sided p-value; R² = coefficient of determination; VIF = variance inflation factor. The T-value indicates the direction of association and the relative weight of a variable in a model; R² indicates the variance explained by the model.

|  |  | Separate models  Univariable (enter) | | | Separate models  Multivariable (enter) | | | | Combined model  Multivariable (backward) | | |
| --- | --- | --- | --- | --- | --- | --- | --- | --- | --- | --- | --- |
|  | n | T | *p* | R² | VIF | T | | p | VIF | T | *p* |
| **Clinical parameters** |  |  |  |  | R² = 0.30 | | | | R² = 0.31 | | |
| Age (years) | 146 | 6.71 | <0.001 | 0.24 | 1.44 | 4.42 | | <0.001 | 1.55 | 3.69 | <0.001 |
| Estimated GFR (ml/min/1.73m²) | 146 | -3.61 | <0.001 | 0.08 | 1.89 | -0.36 | | 0.723 |  |  |  |
| Urea (mg/dl) | 146 | 2.99 | 0.003 | 0.06 | 1.99 | 0.51 | | 0.614 |  |  |  |
| NT-proBNP (pg/ml) | 140 | 1.63 | 0.105 | 0.02 |  |  | |  |  |  |  |
| Mean corpuscular hemoglobin concentration (g/dl) | 146 | -1.83 | 0.069 | 0.02 |  |  | |  |  |  |  |
| Left-atrial volume index (ml/m²) | 143 | 1.01 | 0.313 | 0.01 |  |  | |  |  |  |  |
| Thrombocytes (10³/µl) | 145 | -1.88 | 0.062 | 0.02 |  |  | |  |  |  |  |
| Systolic blood pressure (mmHg) | 145 | 4.74 | <0.001 | 0.14 | 1.22 | 3.29 | | 0.001 | 1.13 | 2.91 | 0.004 |
| 6-minute walking test distance (m) | 138 | -2.77 | 0.006 | 0.05 | 1.40 | 0.79 | | 0.433 |  |  |  |
| Hemoglobin (g/dl) | 146 | -2.34 | 0.020 | 0.04 | 1.34 | -1.12 | | 0.264 |  |  |  |
| Duration of heart failure (years) | 145 | 0.48 | 0.632 | 0.00 |  |  | |  |  |  |  |
| Alanine aminotransferase (U/l) | 145 | -1.26 | 0.210 | 0.10 |  |  | |  |  |  |  |
| Hemoglobin A1c (%) | 145 | 2.97 | 0.004 | 0.06 |  |  | |  |  |  |  |
| Uric acid (mg/dl) | 146 | 1.45 | 0.149 | 0.01 |  |  | |  |  |  |  |
| Creatine kinase (U/l) | 146 | 0.30 | 0.766 | 0.00 |  |  | |  |  |  |  |
| Creatine kinase, muscle-brain (U/l) | 146 | 0.22 | 0.829 | 0.00 |  |  | |  |  |  |  |
| Ferritin (µg/l) | 146 | -0.55 | 0.582 | 0.00 |  |  | |  |  |  |  |
| Basal right ventricular diameter (mm) | 146 | 1.12 | 0.266 | 0.01 |  |  | |  |  |  |  |
| **Serum biomarkers** |  |  |  |  | R² = 0.17 | | | |  |  |  |
| Ln (Neurofilament light chain (pg/ml)) | 146 | 5.34 | <0.001 | 0.17 | 1.36 | | 4.18 | <0.001 | 1.48 | 1.98 | 0.049 |
| Ln (phosphorylated Tau protein (pg/ml)) | 146 | 3.22 | 0.002 | 0.07 | 1.36 | 0.77 | | 0.443 |  |  |  |

**Supplemental Table S12:** Regression analysis of Ln (WMH volume).

GFR = glomerular filtration rate (MDRD formula); *p* = two-sided p-value; R² = coefficient of determination; VIF = variance inflation factor. The T-value indicates the direction of association and the relative weight of a variable in a model; R² indicates the variance explained by the model.

|  |  | Separate models  Univariable (enter) | | | Separate models  Multivariable (enter) | | | | Combined model  Multivariable (backward) | | |
| --- | --- | --- | --- | --- | --- | --- | --- | --- | --- | --- | --- |
|  | n | T | *p* | R² | VIF | T | | p | VIF | T | *p* |
| **Clinical parameters** |  |  |  |  | R² = 0.40 | | | | R² = 0.38 | | |
| Age (years) | 146 | 7.99 | <0.001 | 0.31 | 1.63 | 5.79 | | <0.001 | 1.20 | 7.84 | <0.001 |
| Estimated GFR (ml/min/1.73m²) | 146 | -2.13 | 0.035 | 0.03 | 2.03 | 2.01 | | 0.047 | 1.37 | 2.15 | 0.033 |
| Urea (mg/dl) | 146 | 2.45 | 0.016 | 0.04 | 2.00 | 0.24 | | 0.808 |  |  |  |
| NT-proBNP (pg/ml) | 140 | 4.05 | <0.001 | 0.10 | 1.30 | 3.68 | | <0.001 | 1.18 | 3.85 | <0.001 |
| Mean corpuscular hemoglobin concentration (g/dl) | 146 | -0.89 | 0.373 | 0.01 |  |  | |  |  |  |  |
| Left-atrial volume index (ml/m²) | 143 | 1.84 | 0.068 | 0.02 |  |  | |  |  |  |  |
| Thrombocytes (10³/µl) | 145 | -1.94 | 0.054 | 0.03 |  |  | |  |  |  |  |
| Systolic blood pressure (mmHg) | 145 | 3.80 | <0.001 | 0.09 | 1.26 | 1.25 | | 0.212 |  |  |  |
| 6-minute walking test distance (m) | 138 | -3.27 | 0.001 | 0.07 | 1.42 | -0.16 | | 0.877 |  |  |  |
| Hemoglobin (g/dl) | 146 | -2.03 | 0.044 | 0.03 | 1.41 | -0.15 | | 0.885 |  |  |  |
| Duration of heart failure (years) | 145 | 2.98 | 0.003 | 0.06 | 1.15 | 1.81 | | 0.072 |  |  |  |
| Alanine aminotransferase (U/l) | 145 | -3.00 | 0.003 | 0.06 | 1.25 | -0.08 | | 0.939 |  |  |  |
| Hemoglobin A1c (%) | 145 | 0.74 | 0.458 | 0.00 |  |  | |  |  |  |  |
| Uric acid (mg/dl) | 146 | 1.31 | 0.191 | 0.01 |  |  | |  |  |  |  |
| Creatine kinase (U/l) | 146 | -0.34 | 0.734 | 0.00 |  |  | |  |  |  |  |
| Creatine kinase, muscle-brain (U/l) | 146 | 0.86 | 0.393 | 0.01 |  |  | |  |  |  |  |
| Ferritin (µg/l) | 146 | -0.05 | 0.963 | 0.00 |  |  | |  |  |  |  |
| Basal right ventricular diameter (mm) | 146 | 1.89 | 0.061 | 0.02 |  |  | |  |  |  |  |
| **Serum biomarkers** |  |  |  |  | R² = 0.14 | | | |  |  |  |
| Ln (Neurofilament light chain (pg/ml)) | 146 | 4.37 | <0.001 | 0.18 | 1.36 | | 2.75 | 0.007 |  |  |  |
| Ln (phosphorylated Tau protein (pg/ml)) | 146 | 3.91 | <0.001 | 0.10 | 1.36 | 2.01 | | 0.046 |  |  |  |

**Supplemental Table S13:** Regression analysis of cerebral atrophy.

GFR = glomerular filtration rate (MDRD formula); *p* = two-sided p-value; R² = coefficient of determination; VIF = variance inflation factor. The T-value indicates the direction of association and the relative weight of a variable in a model; R² indicates the variance explained by the model.

|  |  | Separate models  Univariable (enter) | | | Separate models  Multivariable (enter) | | | | Combined model  Multivariable (backward) | | |
| --- | --- | --- | --- | --- | --- | --- | --- | --- | --- | --- | --- |
|  | n | T | *p* | R² | VIF | T | | p | VIF | T | *p* |
| **Clinical parameters** |  |  |  |  | R² = 0.15 | | | | R² = 0.31 | | |
| Age (years) | 146 | 3.93 | <0.001 | 0.10 | 1.34 | 2.46 | | 0.015 | 1.04 | 3.50 | 0.001 |
| Estimated GFR (ml/min/1.73m²) | 146 | -2.16 | 0.033 | 0.03 | 1.44 | 0.40 | | 0.690 |  |  |  |
| Urea (mg/dl) | 146 | 1.92 | 0.057 | 0.03 |  |  | |  |  |  |  |
| NT-proBNP (pg/ml) | 140 | 2.76 | 0.007 | 0.05 | 1.33 | 1.55 | | 0.123 | 1.04 | 2.15 | 0.033 |
| Mean corpuscular hemoglobin concentration (g/dl) | 146 | -1.45 | 0.148 | 0.01 |  |  | |  |  |  |  |
| Left-atrial volume index (ml/m²) | 143 | 2.38 | 0.018 | 0.04 | 1.23 | 0.65 | | 0.519 |  |  |  |
| Thrombocytes (10³/µl) | 145 | -1.65 | 0.100 | 0.02 |  |  | |  |  |  |  |
| Systolic blood pressure (mmHg) | 145 | 0.30 | 0.765 | 0.00 |  |  | |  |  |  |  |
| 6-minute walking test distance (m) | 138 | -3.42 | 0.001 | 0.08 | 1.32 | -1.62 | | 0.180 |  |  |  |
| Hemoglobin (g/dl) | 146 | -2.21 | 0.029 | 0.03 | 1.26 | -0.48 | | 0.635 |  |  |  |
| Duration of heart failure (years) | 145 | 0.22 | 0.829 | 0.00 |  |  | |  |  |  |  |
| Alanine aminotransferase (U/l) | 145 | -1.72 | 0.088 | 0.02 |  |  | |  |  |  |  |
| Hemoglobin A1c (%) | 145 | 0.58 | 0.563 | 0.00 |  |  | |  |  |  |  |
| Uric acid (mg/dl) | 146 | 1.21 | 0.230 | 0.01 |  |  | |  |  |  |  |
| Creatine kinase (U/l) | 146 | -0.63 | 0.529 | 0.00 |  |  | |  |  |  |  |
| Creatine kinase, muscle-brain (U/l) | 146 | 0.48 | 0.632 | 0.00 |  |  | |  |  |  |  |
| Ferritin (µg/l) | 146 | -1.12 | 0.232 | 0.01 |  |  | |  |  |  |  |
| Basal right ventricular diameter (mm) | 146 | 0.98 | 0.329 | 0.01 |  |  | |  |  |  |  |
| **Serum biomarkers** |  |  |  |  | R² = 0.08 | | | |  |  |  |
| Ln (Neurofilament light chain (pg/ml)) | 146 | 2.08 | 0.039 | 0.03 | 1.36 | | 0.44 | 0.657 |  |  |  |
| Ln (phosphorylated Tau protein (pg/ml)) | 146 | 3.41 | 0.001 | 0.08 | 1.36 | 2.69 | | 0.008 |  |  |  |

**Supplemental Table S14:** Regression analysis of hippocampal atrophy.

GFR = glomerular filtration rate (MDRD formula); *p* = two-sided p-value; R² = coefficient of determination; VIF = variance inflation factor. The T-value indicates the direction of association and the relative weight of a variable in a model; R² indicates the variance explained by the model.

**Supplemental figures**

**Figure S1:** **Histograms of crude values and natural log normalized values of NfL and pTau.** Serum concentrations measured by bead-based single molecule immunoassays.

**Supplemental References**

1. Lang RM, Bierig M, Devereux RB, Flachskampf FA, Foster E, Pellikka PA, et al. Recommendations for chamber quantification: a report from the American Society of Echocardiography's Guidelines and Standards Committee and the Chamber Quantification Writing Group, developed in conjunction with the European Association of Echocardiography, a branch of the European Society of Cardiology. J Am Soc Echocardiogr. 2005;18(12):1440-63.

2. Nagueh SF, Appleton CP, Gillebert TC, Marino PN, Oh JK, Smiseth OA, et al. Recommendations for the evaluation of left ventricular diastolic function by echocardiography. J Am Soc Echocardiogr. 2009;22(2):107-33.

3. Zimmermann P, Fimm B. TAP Test battery of Attentional Performance Version 2.2. : Psytest: Herzogenrath.; 2009.

4. Schellig D, Schächtele B. VVM2 Visual and Verbal Memory Test. 2nd extended edition.: Swets Test Services: Frankfurt am Main.; 2009.

5. Wechsler D. Wechsler Memory Scale—Revised manual. : Psychological Corporation: San Antonio, TX.; 1987.

6. Aschenbrenner A, Tucha O, Lange K. RWT Regensburger Wortflüssigkeits-Test. Handanweisung. : Hogrefe Verlag, Göttingen.; 2000.

7. Haid T, Martl C, Schubert F, Wenzl M, Kofler M, Saltuari L. Der HAMASCH 5 Punkt Test. Erste Normierungsergebnisse. Zeitschrift für Neuropsychologie. 2002;13(3):233.

8. Horn W. Performance testing system LPS. 2nd extended edition.1983.

9. Sturm W, Willmes K, Horn W. Performance Testing System 50years+ LPS 50+. Hogrefe, Göttingen. ; 1993.

10. Zigmond AS, Snaith RP. The hospital anxiety and depression scale. Acta Psychiatr Scand. 1983;67(6):361-70.

11. Bjelland I, Dahl AA, Haug TT, Neckelmann D. The validity of the Hospital Anxiety and Depression Scale. An updated literature review. J Psychosom Res. 2002;52(2):69-77.

12. Frey A, Sell R, Homola GA, Malsch C, Kraft P, Gunreben I, et al. Cognitive Deficits and Related Brain Lesions in Patients With Chronic Heart Failure. JACC Heart Fail. 2018;6(7):583-92.

13. Ponikowski P, Voors AA, Anker SD, Bueno H, Cleland JG, Coats AJ, et al. 2016 ESC Guidelines for the diagnosis and treatment of acute and chronic heart failure: The Task Force for the diagnosis and treatment of acute and chronic heart failure of the European Society of Cardiology (ESC). Developed with the special contribution of the Heart Failure Association (HFA) of the ESC. Eur J Heart Fail. 2016;18(8):891-975.
